# Efficacy and Safety of Treatments for Patients With Portal Hypertension and Cirrhosis: A Systematic Review and Bayesian Network Meta-Analysis

**DOI:** 10.3389/fmed.2021.712918

**Published:** 2021-09-03

**Authors:** Qigu Yao, Wenyi Chen, Cuilin Yan, Jiong Yu, Tian'an Jiang, Hongcui Cao

**Affiliations:** ^1^State Key Laboratory for Diagnosis and Treatment of Infectious Diseases, The First Affiliated Hospital, Zhejiang University School of Medicine, Hangzhou, China; ^2^National Clinical Research Center for Infectious Diseases, Hangzhou, China; ^3^Department of Ultrasound, The First Affiliated Hospital, Zhejiang University School of Medicine, Hangzhou, China; ^4^Zhejiang Provincial Key Laboratory for Diagnosis and Treatment of Aging and Physic-Chemical Injury Diseases, Hangzhou, China

**Keywords:** endoscopic therapy, transjugular intrahepatic portosystemic shunt, portal hypertension, liver cirrhosis, network meta-analysis, all-cause rebleeding

## Abstract

**Background and Aims:** Viral hepatitis are one of the main causes of liver cirrhosis. The treatment of portal hypertension caused by liver cirrhosis is difficult and diverse, and the therapeutic effect is unknown. Bayesian network meta-analysis was performed to compare the efficacy and safety of treatments for patients with portal hypertension and cirrhosis, including a transjugular intrahepatic portosystemic shunt (TIPS), endoscopic therapy, surgical therapy and medications.

**Methods:** Eligible articles were searched for in PubMed, Embase, Cochrane Library and Web of Science databases from their inception until June 2020. Using the “gemtc-0.8.4” package in R v.3.6.3 software and the Just Another Gibbs Sampler v.4.2.0 program, network meta-analysis was performed using a random effects model within a Bayesian framework. The odds ratios for all-cause rebleeding, bleeding-related mortality, overall survival (OS), treatment failure and hepatic encephalopathy were determined within the Bayesian framework.

**Results:** Forty randomized controlled trials were identified, including 4,006 adult patients and nine treatment strategies. Our results showed that distal splenorenal shunt and TIPS provided the best control of hemorrhage. Endoscopic variceal ligation with medication resulted in the highest OS rate. Medication alone resulted in poor OS and treatment failure.

**Conclusions:** We performed a systematic comparison of diverse treatments for cirrhotic patients with portal hypertension. Our meta-analysis indicated that a TIPS and distal splenorenal shunt resulted in lower rates of rebleeding than did other therapies. Furthermore, drugs are more suitable for combination therapy than monotherapy.

## Introduction

Esophageal and gastric variceal hemorrhage is a common and life-threatening complication for patients with cirrhosis and portal hypertension (1). In approximately one-third of patients with cirrhosis, hemorrhage may cause early mortality (2). After the first variceal bleeding is addressed, the incidence of rebleeding within 1–2 years is 60–70%, and the mortality rate can be as high as ~20–33% (3, 4). Therefore, monitoring or choosing additional appropriate treatment of hemostasis for the first variceal bleeding may help improve quality of life and prognosis.

Over the past 20 years, various effective treatments for portal hypertension have been developed. Non-selective beta-blockers (β-blockers) remain the cornerstone of bleeding and have been used for more than 30 years (5). In addition, somatostatin and terlipressin are potent splanchnic vasoconstrictors (6). These agents significantly decrease both the hepatic venous pressure gradient and portal-collateral (azygos) blood flow and are used to reduce the risk of bleeding (7). Endoscopic therapy (ET), interventional therapy and surgery are also often used to control hemorrhage. ET involves mainly endoscopic injection sclerotherapy (EIS) and endoscopic variceal ligation (EVL). However, EVL has been shown to result in lower rebleeding, mortality and complication rates compared with EIS (8–10). Transjugular intrahepatic portosystemic shunt (TIPS) is a metal stent that connects the hepatic vein and intrahepatic portal vein to effectively decrease portal pressure and prevent ascites aggravation and rebleeding (11). It is ideal to maintain the portal pressure gradient of the portal and inferior vena cava between 10 and 12 mm Hg (5). Some studies have indicated that the primary unassisted patency rates of polytetrafluorethylene-covered stents are similar to those of surgical shunting (10).

Additionally, several randomized controlled trials (RCTs) have shown that combination therapy may be superior to monotherapy in terms of rebleeding, survival and complication rates (12). Argonz et al. reported increased recurrence of bleeding in the EVL group compared with the EVL plus EIS group (31.7 vs. 23%) (13). Similarly, a meta-analysis found that EVL plus nadolol or sucralfate decreased the risk of rebleeding compared with EVL alone. However, Puente et al. noted that a reduction in rebleeding did not improve survival (14).

To use existing study-level data to assess the relative effectiveness of active interventions in cirrhotic patients with a history of hemorrhage, we performed a network meta-analysis of RCTs that included rebleeding and mortality as outcomes. The purpose of this meta-analysis is to provide guidance for clinical policymakers regarding the safety and efficacy of TIPS, EVL, EIS, medication and combinations of these treatments in terms of the 1-, 2-, and 3-year rebleeding and overall survival (OS) rates, treatment failure, bleeding-related mortality, and HE.

## Materials and Methods

### Search Strategy

Two researchers independently screened the titles and abstracts of the articles in terms of the selection criteria. The literature search was performed in various electronic databases (PubMed, Web of Science, MEDLINE, Embase and Cochrane Library) from their inception to June 2020. A combination of free-text terms and medical subject heading terms were used for the subject search, as follows: “liver cirrhosis,” “variceal hemorrhage,” “variceal rebleeding,” “transjugular intrahepatic portosystemic shunt,” “balloon-occluded retrograde transvenous obliteration,” “endoscopic therapy,” “beta-blocker,” and “surgery.” The article type was restricted to randomized controlled trials.

### Study Selection

Studies were considered eligible for inclusion if they fulfilled the following criteria: (1) focused on treatments for patients with cirrhosis and portal hypertension; (2) compared at least two factors among TIPS, EVL, EIS, medication, or combination therapies; and (3) included rebleeding as a primary endpoint. The exclusion criteria were as follows: (1) not written in English; (2) non-clinical article, such as a case report, letter, basic research study or systematic review; (3) lack of sufficient or qualified data; (4) published before 2,000 or included <20 participants per group.

### Data Extraction and Quality Assessment

Two researchers independently extracted the data, and a third researcher was consulted to reach a majority decision when needed. The following information was summarized. (1) The authors' names, year of publication, treatment group, country of study, number of patients and follow-up time. (2) Clinical outcomes including all-cause rebleeding; 1-, 2-, and 3-year rebleeding rates; treatment failure; bleeding-related mortality; 1-, 2-, and 3-year OS rates; and HE. Otherwise, treatment failure was defined as the occurrence of two or more episodes of recurrent bleeding or switching to an alternative treatment. This meta-analysis was conducted in accordance with the guidelines of the Preferred Reporting Items for Systematic Review and Meta-Analysis Protocols 2015 statement (15). The methodological quality of the RCTs was evaluated using the Cochrane Collaboration tool (16).

### Statistical Analysis

Using the “gemtc” package in R 3.6.3 software, the Markov chain Monte Carlo method was applied to perform Bayesian network meta-analysis (17). This method combines both direct and indirect evidence for any given pair of management strategies and a particular endpoint. The mtc.run function was applied to generate samples, and we set 5,000 simulations for each chain as the “burn-in” period, yielding 20,000 iterations to obtain the odds ratios (ORs) for the model parameters based on three Markov chains. Rank probabilities were calculated to obtain the hierarchical position of each treatment, and a plot of rank probabilities was created using the “gemtc” package (18). Brooks–Gelman–Rubin plots, trace plots and density plots were used to assess model convergence (19).

The mtc.anohe command of the “gemtc” package was used to evaluate global heterogeneity. To ensure reliability, a sensitivity analysis was performed by removal of each trial. Begg's test and Egger's test were applied using a *P* < 0.1 threshold of significance for testing publication bias.

## Results

### Eligible Studies and Characteristics

**Figure 1:**
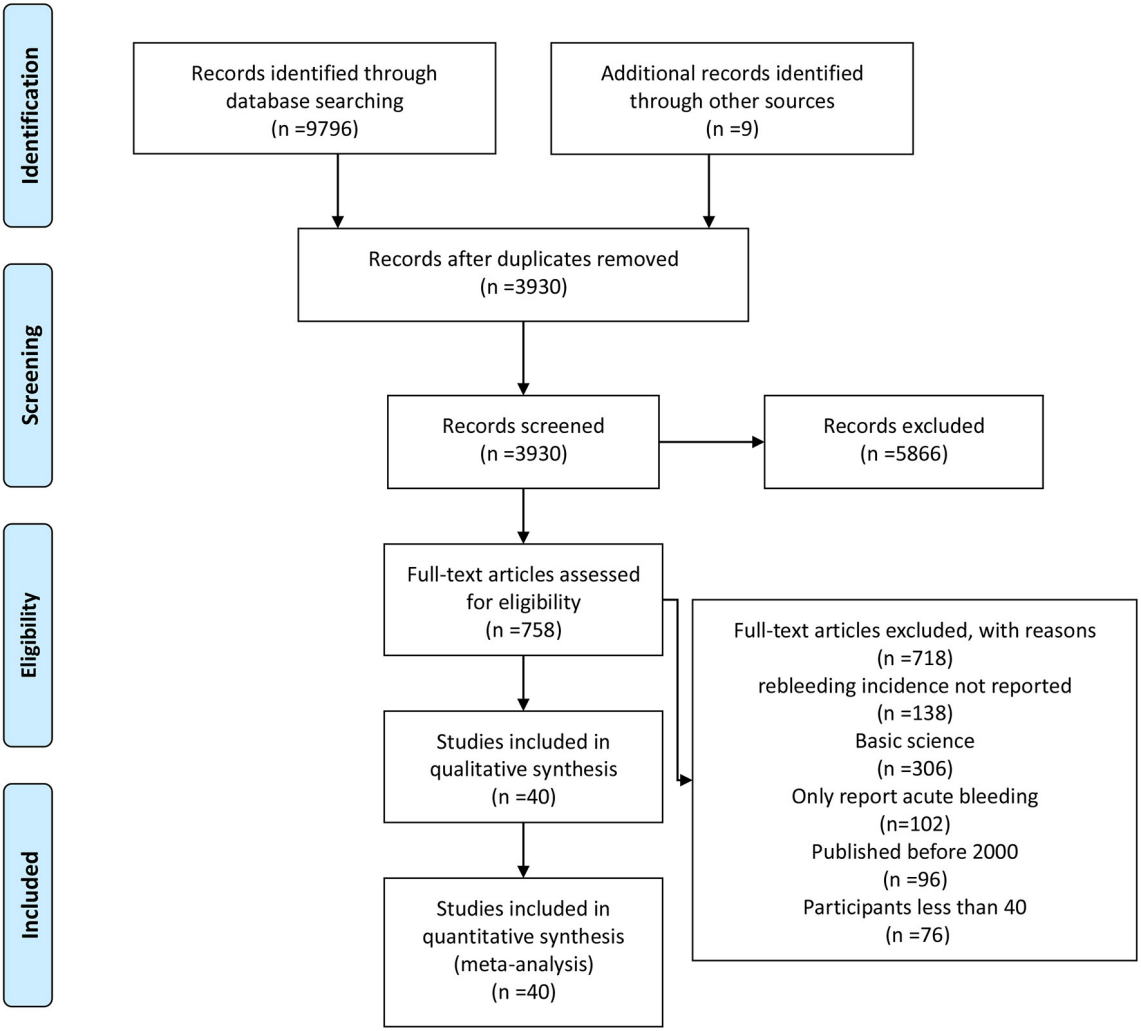
Flow chart of the study selection.

The literature search generated 9,805 relevant clinical records. After screening titles and abstracts, removing duplicates and assessing eligibility, 5,866 articles were excluded; the remaining articles were subjected to full text review. Finally, 40 RCTs including a total of 4,006 patients met the inclusion criteria and were selected for the meta-analysis. A flow chart of the detailed screening process can be found in Figure 1.

### Study Characteristics and Quality

The basic characteristics of the selected studies are summarized in Table 1. In total, 4,006 patients were enrolled in nine different treatment strategies. The 40 studies included 12 from China, five from India, one from Japan, one from Pakistan, one from Mexico, two from Argentina, one from Canada, one from the USA, one Croatia, one Brazil, one from Italy, four from Egypt, three from Germany, four from Spain, and two from Greece. Thus, 19 studies were performed in Asia, 11 in Europe, six in America, and four in Africa. Seventeen of the studies had a mean follow up time of >2 years. Medications included propranolol, nadolol, octreotide, terlipressin, and isosorbide-5-mononitrate; ET included EIS, EVL, and endoscopic tissue adhesive (ETA) injection. There were two major types of surgical shunts: portacaval and distal splenorenal shunts. All studies were two-arm trials, except for Harras consisting of three arms: EIS, EVL, and EIS + EVL. Detailed results of the bias assessment are shown in Supplementary Table 1.

**Table 1:** Study characteristics.

**References**	**Country**	**Sarin classification**	**Etiology of cirrhosis, alcohol/** **Hepatitis/others**	**Male/female**	**Treatment**	**Number of patients**	**Child-Pugh class (A/B/C)**	**Child-Pugh score**	**Follow-up time mean (range or SD)**
Argonz et al. (13)	Argentina	G2/G3: 27,14	20/12/9	32/9	EVL	41	14/23/4	NA	337 ± 43.4 days
		G2/G3: 22,17	24/6/9	30/9	EVL+EIS	39	11/26/2	NA	386 ± 40.1 days
Hou et al. (9)	China	F3/F2+F: 151,19	13/44/13	57/13	EIS	70	17/34/19	8 ± 1.9	63.4 ± 11.6 months
		F3/ F2+F1: 57,14	11/41/19	56/15	EVL	71	20/26/25	8.4 ± 2.4	60.1 ± 17.5 months
Lo et al. (20)	China	F2/F3: 27,35	20/41/1	49/13	EVL	62	12/28/22	NA	21 months
		F2/F3: 24,36	17/41/2	45/15	EVL+ Drug (Nadolol)	60	13/30/19	NA	21 months
Orozco et al. (21)	Mexico	NA	NA	NA	Drug (Nadolol)	40	22/14/4	NA	45 months
		NA	NA	NA	EIS	46	21/17/8	NA	45 months
Villanueva et al. (22)	Spain	G1/G2/G3: 1,49,22	30/26/13	47/25	EVL	72	11/43/18	8.4 ± 1.9	21 months
		G1/G2/G3: 2,41,29	33/24/10	43/29	Drug (Nadolol+ ISMN)	72	19/39/14	7.9 ± 1.9	21 months
Pomier-Layrargues et al. (23)	Canada	NA	24/5/10	27/12	EVL	39	NA	9.8 ± 1.6	48.5 months
		NA	25/4/12	29/12	TIPS	41	NA	9.6 ± 1.6	22.6 months
Hou et al. (24)	China	NA	NA	31/16	EVL	47	14/17/16	8.2 ± 2.3	11.6 ± 6 months
		NA	NA	36/11	EVL+EIS	47	11/23/13	8 ± 2.1	11.1 ± 5.9 months
Cheng et al. (25)	China	F1/F2/F3: 8,21,13	NA	30/12	EVL	42	14/17/11	NA	NA
		F1/F2/F3: 11,23,10	NA	29/15	EIS+EVL	44	18/14/12	NA	NA
Narahara et al. (26)	Japan	NA	9/24/5	32/6	TIPS	38	NA	6.8 ± 0.3	1,116 ± 92 days
		NA	17/20/3	30/10	EIS	40	NA	7.4 ± 0.3	1,047 ± 102 days
Sauer et al. (27)	Germany	MG 2.4: 43	29/9/5	27/16	TIPS	43	15/16/12	7.9 ± 2.1	4.1 ± 0.26 months
		MG 2.6: 42	24/12/6	23/19	EVL	42	10/19/13	8.2 ± 2.0	3.6 ± 0.25 months
Gülberg et al. (28)	Germany	NA	22/3/3	20/8	TIPS	28	11/15/2	NA	21.6 months
		NA	23/3/0	19/7	EVL	26	10/12/4	NA	24.0 months
Escorsell et al. (29)	Spain	NA	25/NA/19	35/9	Drug (Propranolol+ ISMN)	44	0/28/16	6.3 ± 1.3	15.4 ± 10.3 months
		NA	24/NA/23	33/14	TIPS	47	0/30/17	7.0 ± 1.4	14.4 ± 9.6 months
Viazis et al. (10)	Greece	S/M /L: 9,17,10	15/15/6	21/15	EVL	36	4/18/14	NA	49.6 ± 9.7 days
		S/M/L: 10,15,12	13/17/7	20/17	EIS	37	6/16/15	NA	58.6 ± 10.4 days
Avgerinos et al. (8)	Greece	G1/G2/G3: 3,13,9	17/8/0	19/6	EVL	25	6/8/11	9.4 ± 2.8	42 days
		G1/G2/G3: 3,12,10	15/7/3	20/5	EIS	25	7/6/12	9.2 ± 2.96	42 days
Schepke et al. (30)	Germany	NA	40/22/13	50/25	EVL	75	34/31/10	7.3 ± 1.8	34.4 ± 18.9 months
		NA	38/25/14	54/23	Drug (Propranolol)	77	37/31/9	7.0 ± 1.9	34.4 ± 18.9 months
Peña et al. (31)	Spain	II/III/IV: 7,17,13	26/8/3	27/10	EVL	37	6/20/11	NA	15 ± 8 months
		II/III/IV: 10,26,7	27/12/4	33/10	EVL+ Drug (Nadolol)	43	6/25/12	NA	17.5 ± 7.8 months
Sarin et al. (32)	India	NA	18/25/10	51/20	EVL	71	35/26/10	6.9 ± 2.0	12.4 ± 9.7 months
		NA	15/23/8	45/21	Drug (β-blocker+ ISMN)	66	27/28/11	7.2 ± 1.9	11.1 ± 7.9 months
Shah et al. (33)	Pakistan	NA	2/49/NA	32/19	EIS +Drug (Octreotide)	51	9/31/11	NA	24 months
		NA	2/52/NA	36/18	EIS	54	7/33/14	NA	24 months
Zargar et al. (34)	India	G2/3/4: 1,8,27	NA	22/14	EIS	36	NA	NA	NA
		G2/3/4: 2,6,29	NA	24/13	EVL	37	NA	NA	NA
Chen et al. (35)	China	F1/F2/F3: 5,38,19	24/30/8	43/19	EVL	62	13/31/18	8.3 ± 2.0	NA
		F1/F2/F3: 2,41,20	29/29/5	52/11	SMT	63	18/27/18	8.3 ± 2.3	NA
Santambrogio et al. (36)	Italy	NA	14/NA/NA	27/13	DSRS	40	19/21/ NA	NA	109.0 ± 58 months
		NA	26/NA/NA	33/7	EIS	40	11/29/ NA	NA	87.0 ± 61 months
Romero et al. (37)	Argentina	NA	30/8/19	37/20	Drug (Nadolol+ ISMN)	57	40/44/16	7.0 ± 1.9	11.5 months
		NA	24/7/21	35/17	EVL	52	32/58/10	7.0 ± 1.6	12 months
Henderson et al. (38)	America	NA	NA	42/31	DSRS	73	41/32/NA	6.4 ± 1.1	45 months
		NA	NA	44/23	TIPS	67	39/28/NA	6.3 ± 1.0	45 months
Tan et al. (39)	China	G1/G2/IGV1: 11,5,5	3/32/13	34/14	EVL	48	12/25/11	8.10 ± 2.22	610.6 ± 603.04 days
		G1/G2/IGV1: 7,3,1	2/31/16	35/14	ETA	49	13/26/10	7.96 ± 1.86	680.7 ± 710.54 days
Lo et al. (40)	China	G1/G2: 19,14	NA	25/10	TIPS	35	9/20/6	7.8 ± 1.8	33 months
		G1/G2: 17,19	NA	28/9	ETA	37	12/19/6	7.6 ± 1.7	32 months
Morales et al. (41)	Brazil	NA	9/17/14	27/13	EIS+ Drug (Octreotide)	40	4/12/24	6.8 ± 0.3	14 months
		NA	2/11/15	18/10	EIS	28	7/11/10	NA	14 months
Amin et al. (42)	Egypt	NA	NA/68/NA	53/22	EVL	75	20/40/15	NA	NA
		NA	NA/65/NA	55/20	ETA	75	15/32/28	NA	NA
Lo et al. (43)	China	NA	16/32/12	46/14	EVL	60	13/35/12	8.0 ± 1.5	82 months
		NA	22/32/7	47/14	Drug (Nadolol+ ISMN)	61	13/35/13	7.8 ± 1.6	81 months
Kumar et al. (44)	India	MG 3.1: 88	33/13/30	75/13	EVL+ Drug (Propranolol+ ISMN)	88	35/31/10	7.3 ± 2.0	15 ± 12 months
		MG 3.2: 89	30/20/25	78/11	EVL	89	26/34/15	7.8 ± 2.1	15 ± 11 months
Lo et al. (12)	China	NA	17/25/4	41/5	Drug (Terlipressin)	46	14/25/7	7.7 ± 1.8	42 days
		NA	15/28/4	36/11	EVL+ Drug (Terlipressin)	47	13/20/14	8.1 ± 1.8	42 days
García*-*Pagán et al. (45)	Spain	NA	42/18/18	53/25	Drug (β-blocker+ ISMN)	78	18/42/18	8.1 ± 1.8	15 months
		NA	39/25/16	65/15	EVL+ Drug (β-blocker+ ISMN)	80	16/46/18	8.2 ± 1.8	15 months
Sarin et al. (46)	India	II/III/IV: 12,20,19	NA	38/13	EVL	51	NA	5 (5–9)	23 months
		II/III/IV: 17,19,14	NA	32/18	Drug (Propranolol)	50	NA	5 (5–7)	23 months
Mishra et al. (47)	India	NA	12/NA/NA	19/14	ETA	33	4/12/17	9 (6–12)	26 months
		NA	11/NA/NA	26/8	Drug (β-blocker)	34	5/13/16	9 (6–12)	26 months
Harras et al. (48)	Egypt	G1/G2/G3: 0,2,48	NA	NA	EIS	50	16/29/5	NA	17.8 ± 4.85 months
		G1/G2/G3: 0,0,50	NA	NA	EVL	50	14/32/4	NA	17.8 ± 4.85 months
		G1/G2/G3: 0,1,49	NA	NA	EIS+EVL	50	12/36/2	NA	17.8 ± 4.85 months
Ljubicić et al. (49)	Croatia	NA	NA	16/6	ETA	22	4/9/9	NA	60 months
		NA	NA	15/6	EVL	21	8/9/4	NA	60 months
Kong et al. (50)	China	f2/f3: 10,10	2/10/6	14/6	EVL	20	9/11/NA	NA	48 months
		f2/f3: 9,11	1/10/6	9/9	EVL+EIS	18	8/10/NA	NA	48 months
Ali et al. (51)	China	G2/G3: 11,53	4/37/23	51/13	EIS	64	20/33/11	NA	24 months
		G2/G3: 8,52	3/39/18	45/15	EVL	60	22/29/9	NA	24 months
Lv et al. (52)	China	NA	1/21/2	13/11	TIPS	24	9/13/2	7 (6–8)	30 months
		NA	0/22/3	16/9	EVL+ Drug (Terlipressin or Somatostatin)	25	10/14/1	7 (6–8)	30 months
Mansour et al. (53)	Egypt	G1/G2: 49,11	NA/56/4	34/26	EVL	60	8/20/32	NA	12 months
		G1/G2: 45,15	NA/60/0	44/16	EVL+EIS	60	14/22/24	NA	12 months
Elsebaey et al. (54)	Egypt	F1/F2/F3: 2,22,32	NA	42/14	EIS	56	13/23/20	NA	20 months
		F1/F2/F3: 4,24,29	NA	39/18	ETA	57	15/24/18	NA	20 months

*G, Grade; Grade 1, visible only during 1 phase of respiration or on performance of valsalva maneuver; Grade 2, visible during both phases of respiration; Grade 3, 3–6 mm; Grade 4, >6 mm; S, small: V arix is flush with the wall of the oesphagus; M, medium: Protrusion of varix no further than halfway to the center; L, large: protrusion more than half way to the center of the lumen; F, varicies; F1, straight, small-caliber varices; F2, moderately enlarged, beady varices; F3, markedly enlarged, nodular or tumor-shaped varices. DSRS, distal splenorenal shunt; TIPS, transjugular intrahepatic portosystemic shunt; EIS, endoscopic injection sclerotherapy; EVL, endoscopic variceal ligation; ETA, endoscopic tissue adhesion; ISMN, isosorbide mononitrate; MG, mean grade; NA, not available*.

### Network Structure Diagrams

**Figure 2:**
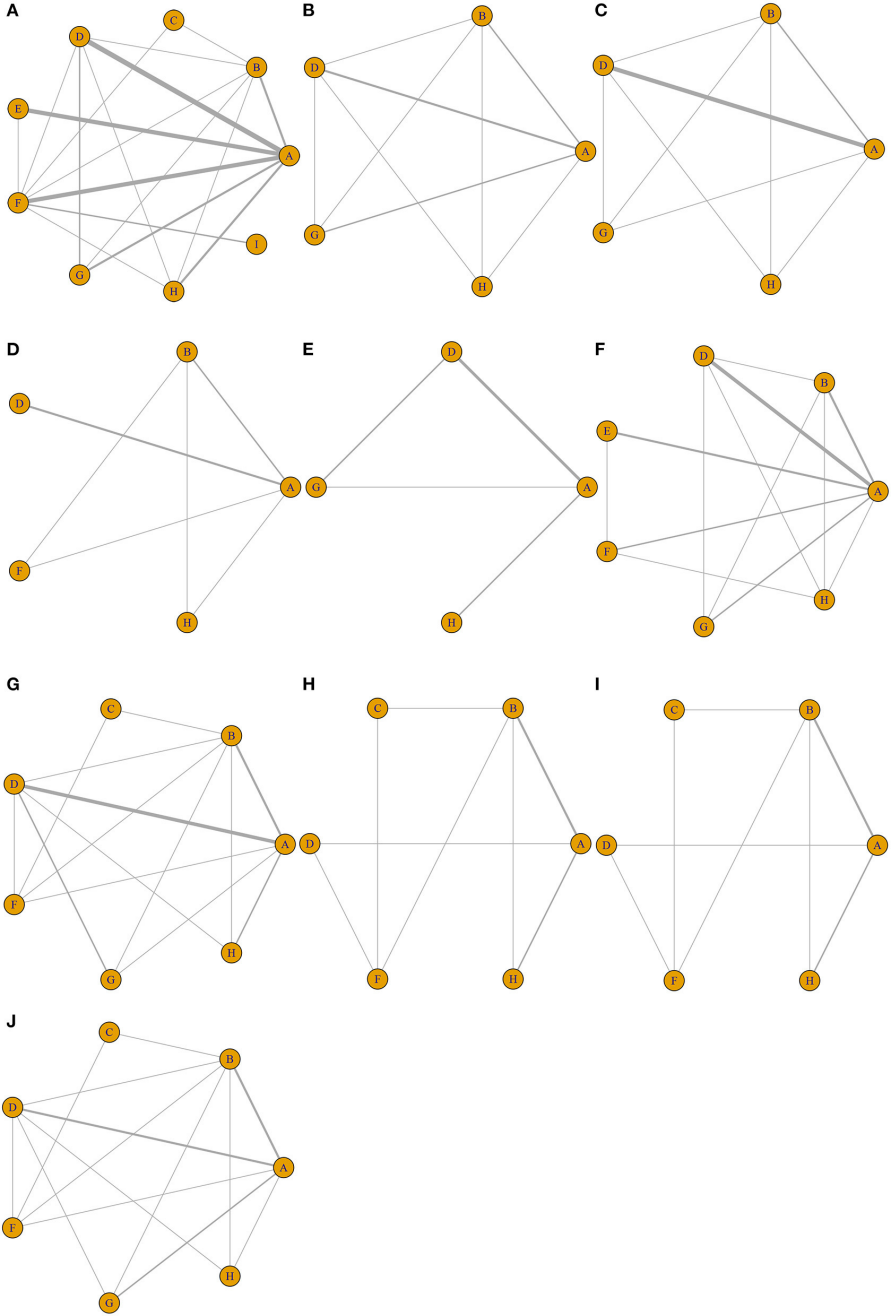
Network structure diagrams. As shown in the figure, the thickness of the lines is proportional to the number of comparisons, and the diameter of the circles is proportional to the number of treatments included in the meta-analysis. **(A)** All-cause rebleeding. Rebleeding at **(B)** 1 year, **(C)** 2 years, and **(D)** 3 years. **(E)** Treatment failure. **(F)** Bleeding-related mortality. OS at **(G)** 1 year, **(H)** 2 years, and **(I)** 3 years. **(J)** Hepatic encephalopathy.

Nine different therapeutic strategies were included among the trials: EVL, TIPS, distal splenorenal shunt (DSRS), medication, EVL + EIS, EIS, EVL + medication, ETA, and EIS + medication. Network structure diagrams were applied to depict the direct associations among the treatment strategies. The thickness of the lines is proportional to the number of comparisons, and the diameter of the circles is proportional to the number of treatments included in the meta-analysis. All diagrams are presented in Figure 2.

### Brooks–Gelman–Rubin Diagnostic Plot, Density Plot, and Trace Plot

Brooks–Gelman–Rubin diagnostic plots, trace plots and density plots were obtained to assess the convergence of our model. As suggested by Brooks and Gelman (55), the model was considered to be well-fitted if the curves of the plots were consistent and stable, and if the potential scale reduction factor was close to 1.0. For trace plots, each Markov Chain Monte Carlo chain achieved stable fusion from the beginning, and the overlapping area accounted for the majority of chain fluctuation in the subsequent calculations. The fluctuation of single chains could not be recognized by the naked eye, and therefore the degree of convergence was considered satisfactory, as shown in Supplementary Figure 1. In the density diagram, the bandwidth tended to be zero and stable, and a smooth curve that conformed to the normal distribution indicated that the model had good convergence (Supplementary Figure 1). Furthermore, the potential scale reduction factor for each analysis was close to 1.0 in the Brooks–Gelman–Rubin diagnostic plot, as shown in Supplementary Figure 2 and Supplementary Table 2.

### All-Cause Rebleeding

**Figure 3:**
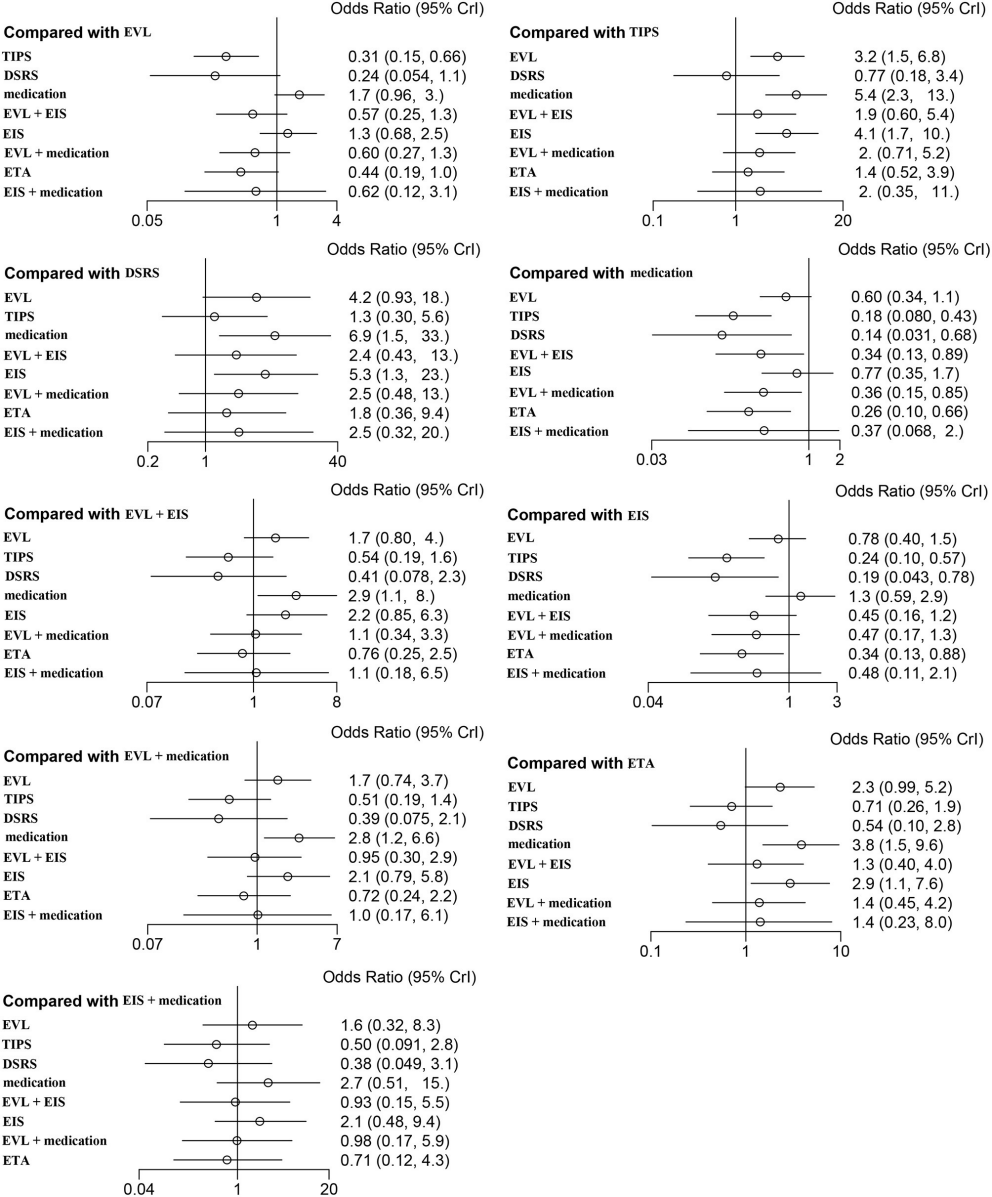
Forest plot of the odds ratios for all-cause rebleeding based on different pairwise comparisons. EVL, endoscopic variceal ligation; TIPS, transjugular intrahepatic portosystemic shunt; DSRS, distal splenorenal shunt; EIS, endoscopic injection sclerotherapy; ETA, endoscopic tissue.

**Figure 4:**
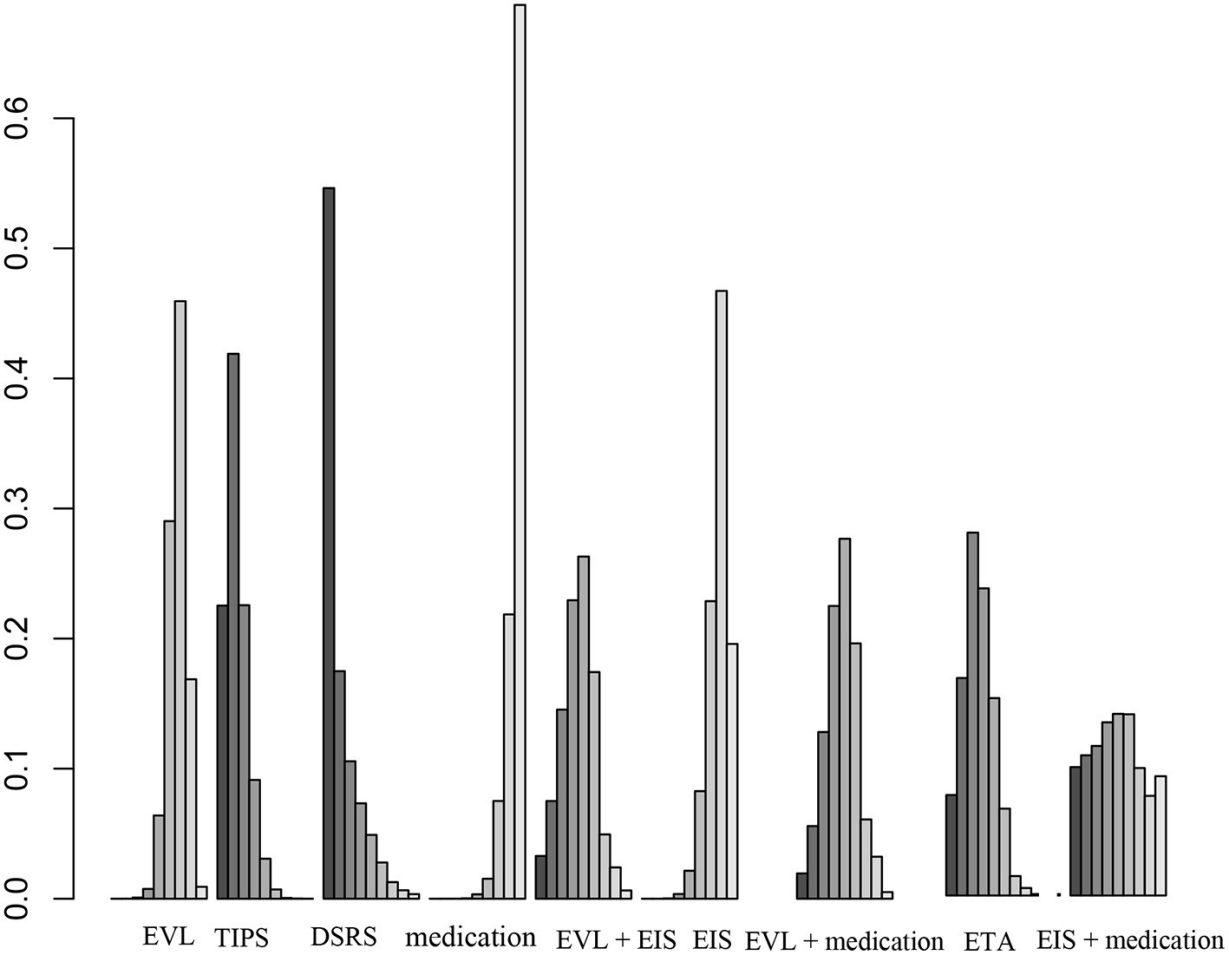
Ranking of therapies based on all-cause rebleeding. EVL, endoscopic variceal ligation; TIPS, transjugular intrahepatic portosystemic shunt; DSRS, distal splenorenal shunt; EIS, endoscopic injection sclerotherapy; ETA, endoscopic tissue.

Forty studies, including 4,006 patients with nine therapeutic schedules, reported all-cause rebleeding. There were many significant differences among the therapeutic schedules, such as TIPS vs. EVL (OR 0.31, 95% CI 0.15–0.66), medication vs. TIPS (OR 5.4, 95% CI 2.3–13.0), EIS vs. TIPS (OR 4.1, 95% CI 1.7–10.), medication vs. DSRS (OR 1.3, 95% CI 0.30–5.6), EIS vs. DSRS (OR 5.3, 95% CI 1.3–23.), medication vs. EVL + EIS (OR 2.9, 95% CI 1.1–8), EVL + medication vs. medication alone (OR 0.36, 95% CI 0.15–0.85), ETA vs. medication (OR 0.26, 95% CI 0.10–0.66), and ETA vs. EIS (OR 0.34, 95% CI 0.13–0.88) (Figure 3). Subgroup analyses of rebleeding occurring at 1, 2, and 3 years are summarized in Supplementary Figures 3–5. Compared with TIPS, EVL (OR 6.1, 95% CI 2.2–18.0; OR 6.6, 95% CI 2.5–18.0; and OR 5.0, 95% CI 0.9–28.0 at 1, 2, and 3 years, respectively) and medication (OR 4.9, 95% CI 1.7–17.0; OR 7.9, 95% CI 2.9–23.0; and OR 11, 95% CI 1.0–120.0 at 1, 2, and 3 years, respectively) had higher rates of rebleeding in all years. In descending order, all-cause rebleeding was best controlled by DSRS, TIPS, EIS + medication, ETA, EVL + medication, EVL + EIS, EVL, EIS, and medication alone (Figure 4). In order of decreasing efficacy, rebleeding at 1, 2, and 3 years were best controlled by TIPS, ETA, EVL + medication/EIS, EVL, and medication alone (Supplementary Figure 6).

### Bleeding-Related Mortality

Twenty-one articles including seven different treatments (EVL, TIPS, medication, EVL + EIS, EIS, EVL + medication, and ETA) were used in the analysis of bleeding-related mortality. No significant results were identified regarding the previously discussed treatments. Our results showed that EVL (OR 5.0, 95% CI 0.6–100.0), TIPS (OR 2.6, 95% CI 0.2–51.0), medication alone (OR 5.0, 95% CI 0.5–100.0), EIS (OR 5.4, 95% CI 0.5–130.0), EVL + medication (OR 2.4, 95% CI 0.2–54.0), and ETA (OR 2.3, 95% CI 0.2–50.0) were associated with a relatively high rate of rebleeding compared with EVL + EIS (Supplementary Figure 7). As indicated in the cumulative ranking, EVL + EIS ranked most favorably among the treatments (Supplementary Figure 8).

### Overall Survival (OS)

**Figure 5:**
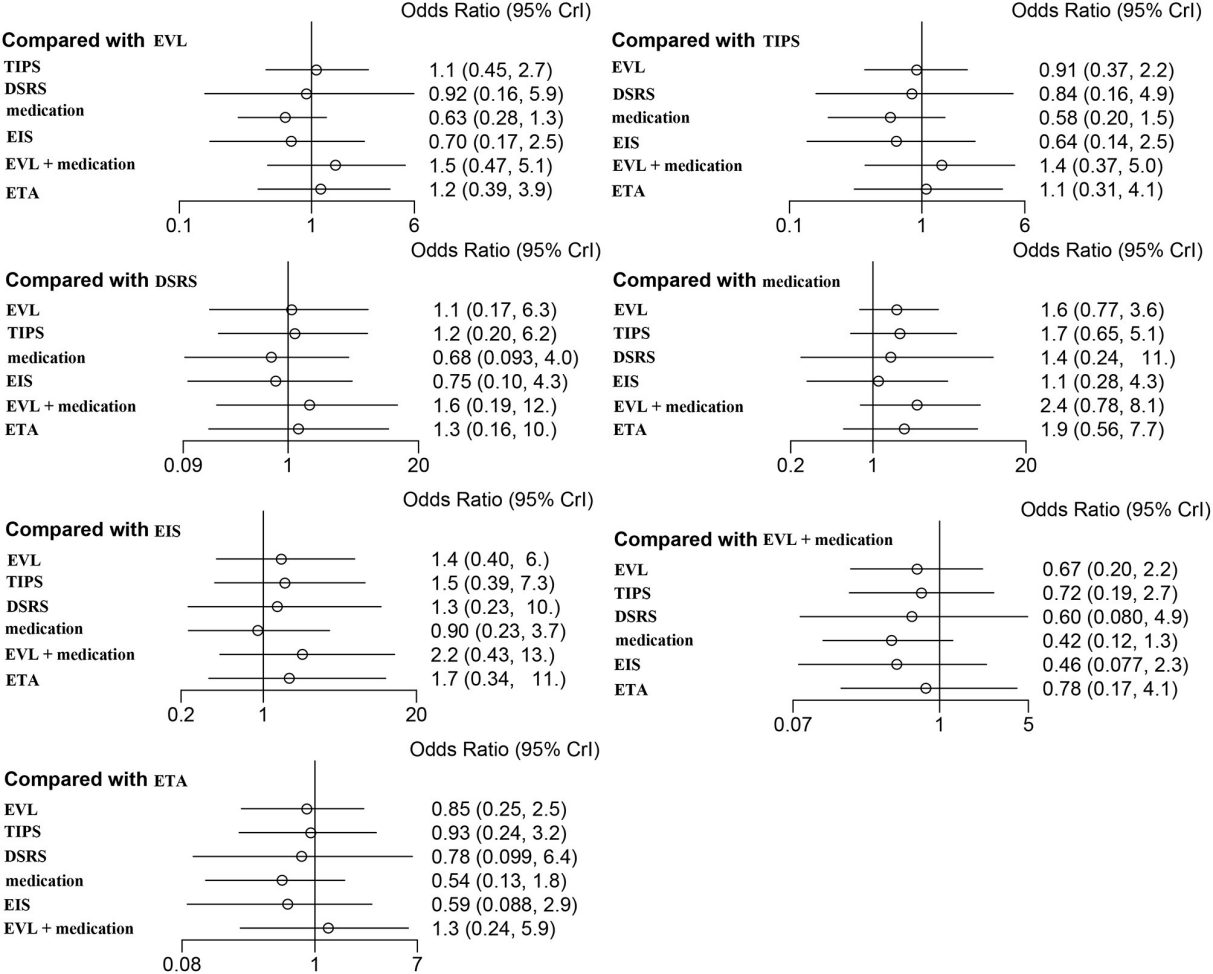
Forest plot of the odds ratios for 1-year OS based on different pairwise comparisons. EVL, endoscopic variceal ligation; TIPS, transjugular intrahepatic portosystemic shunt; DSRS, distal splenorenal shunt; EIS, endoscopic injection sclerotherapy; ETA, endoscopic tissue.

**Figure 6:**
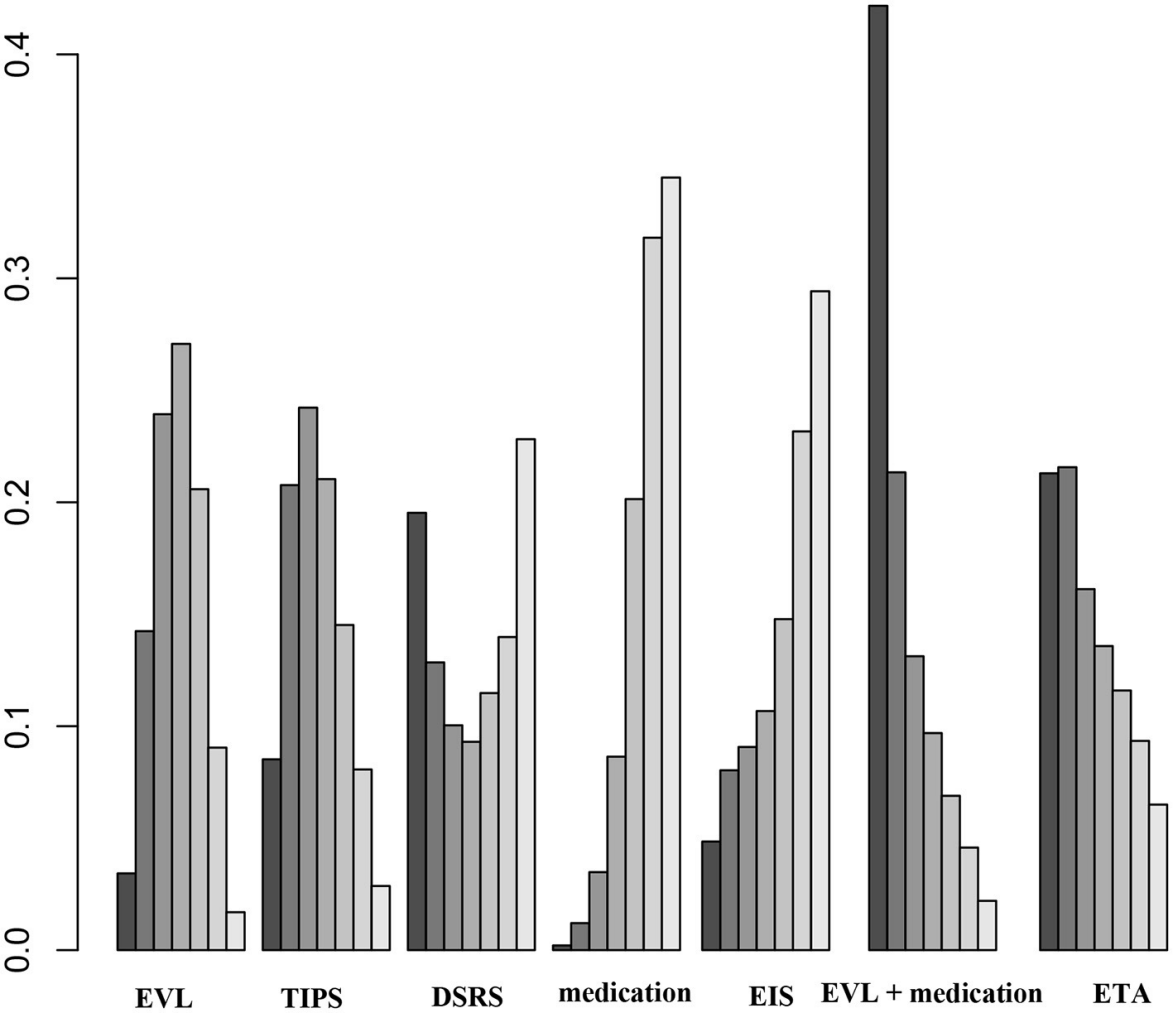
Ranking of 1-year OS among the different therapies. EVL, endoscopic variceal ligation; TIPS, transjugular intrahepatic portosystemic shunt; DSRS, distal splenorenal shunt; EIS, endoscopic injection sclerotherapy; ETA, endoscopic tissue.

Twenty-one of the selected studies assessed the 1-year OS rate in a total of 2,163 patients with cirrhosis. Eleven of the studies assessed 2- and 3-year OS rates in a total of 967 cirrhosis patients. Our results showed that TIPS (OR 1.1, 95% CI 0.5–2.7), DSRS (OR, 0.9 95% CI 0.2–5.9), medication (OR 0.6, 95% CI 0.3–1.3), EIS (OR 0.7, 95% CI 0.2–2.5), EVL + medication (OR 1.5, 95% CI 0.5–5.1), and ETA (OR 1.2, 95% CI 0.4–3.9) did not differ significantly from EVL in terms of 1-year OS (Figure 5). There was also no significant difference among the other treatment methods (Figure 5). EVL, TIPS, DSRS, medication, EIS and ETA offered no significant benefit in terms of the 2- or 3-year OS rate (Supplementary Figures 9, 10). Rank probability analysis showed that medication, EIS, DSRS, TIPS, EVL, ETA, and EVL + medication ranked from worst to best in terms of 1-year OS, and that medication, TIPS, EVL, ETA, DSRS, and EIS ranked from worst to best in terms of 3-year OS (Figure 6 and Supplementary Figures 11, 12).

### Treatment Failure

The incidence of treatment failure was examined in nine direct comparisons and just four different treatments among 1,099 patients. The incidence of treatment failure is shown in Supplementary Figure 13. Compared with EVL, medication (OR 1.3, 95% CI 0.4–5.7), EVL + medication (OR 0.3, 95% CI 0.0–2.4), and ETA (OR 1.2, 95% CI 0.1–13.0) were not significantly associated with treatment failure. When other treatments were compared, the results were similar. We created a rank probability plot, which showed that EVL + medication had the lowest rate of treatment failure (Supplementary Figure 14).

### Hepatic Encephalopathy

**Figure 7:**
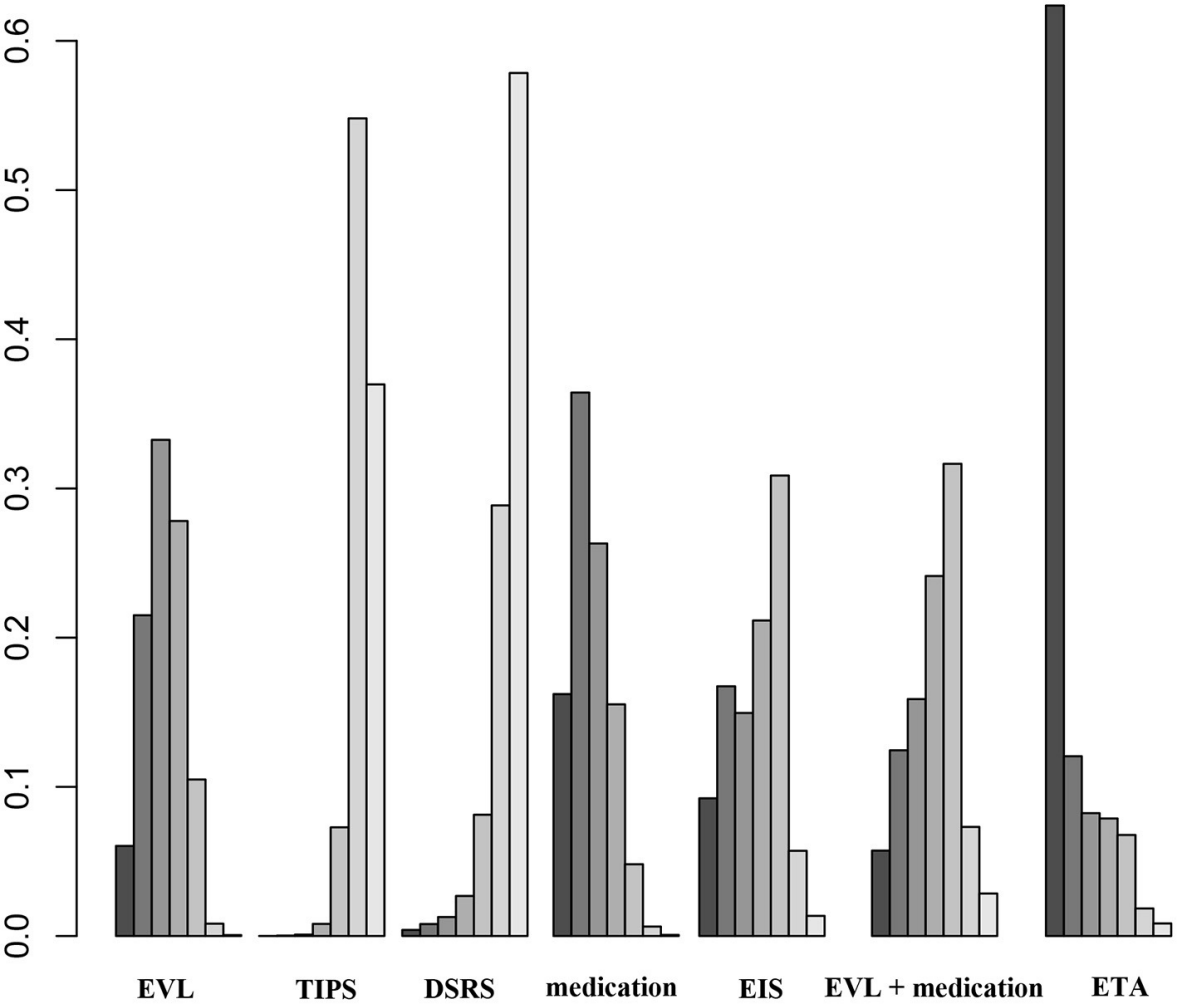
Ranking of the odds ratios for HE based on different pairwise comparisons. EVL, endoscopic variceal ligation; TIPS, transjugular intrahepatic portosystemic shunt; DSRS, distal splenorenal shunt; EIS, endoscopic injection sclerotherapy; ETA, endoscopic tissue.

A total of 1,956 patients experienced hepatic encephalopathy in trials that included 19 direct comparisons and seven different treatments. As shown in Figure 7, DSRS and TIPS were associated with a significantly higher incidence of hepatic encephalopathy compared with EVL, medication, EIS, EVL + medication, and ETA. The rank probability analysis confirmed this finding (Supplementary Figure 15).

### Sensitivity Analysis and Publication Bias

Sensitivity analysis was performed by excluding several studies. The results were consistent with those of the primary meta-analysis. Begg's and Egger's tests showed that no clear publication bias existed (*P* > 0.1).

## Discussion

Abundant research has shown that variceal bleeding and rebleeding are among the most serious complications of portal hypertension in patients with cirrhosis because of a severe impact on prognosis (11, 56). Treating patients based on their individual risk of portal hypertension-related bleeding undoubtedly affects the prognosis. ET, a classic treatment that has been routinely used for 30–40 years, plays a pivotal role in the management of variceal bleeding and rebleeding (57). However, ET is effective for only a short time because portal pressure and blood flow remain unchanged, and varices frequently recur (in ~50% of cases within 2 years). β-blockers, such as propranolol, timolol, nadolol and carvedilol, decrease cardiac output by β1 adrenergic receptors and reduce splanchnic blood flow by β2 receptors. None of these medications are clearly more effective than others; their usage is driven by doctors' recommendations and patient compliance (58, 59). It has been reported that combination therapy with β-blockers and EVL is significantly more effective than either EVL or medication alone in preventing recurrent hemorrhage (46, 60). In addition, adding low-dose isosorbide-5-mononitrate to β-blockers has been shown to provide a greater portal pressure-reducing effect than β-blockers alone (37, 61). As shown in our network diagram, many studies have directly compared the above treatments, but several treatments have not been compared directly (e.g., EIS + medication vs. DSRS, EIS + medication vs. TIPS, etc.). We conducted our network meta-analysis to address these gaps and provide further guidance for clinical practice.

Our network meta-analysis included 40 RCTs that were conducted within the past 20 years and compared rates of rebleeding, treatment failure, OS and HE due to variceal hemorrhage. A total of 4,006 patients were treated with nine therapeutic methods, including vasoactive medications, DSRS, EVL, EIS, ETA, and combination therapies.

We found that TIPS and DSRS were associated with a lower likelihood of variceal rebleeding compared with ET or medication, either alone or combined. However, TIPS and DSRS were also associated with a higher rate of HE, which is consistent with the American Association for the Study of Liver Diseases practice guidelines (62). Patients experienced a peak incidence of ascites early after DSRS placement (~10% within the 1st month) (38, 63). Conversely, there was a high rate of ascites in the TIPS group at later follow-up time points. TIPS is preferred over DSRS for patients with poor liver function after ineffective conservative therapy and ET. Otherwise, both DSRS and TIPS appear to offer equivalent outcomes (59). According to the AALSD and Baveno guidelines, although TIPS and DSRS are effective in controlling rebleeding, we need to discuss treatment indications the issue of critical liver reserve. TIPS is only recommended for early intervention (72 h) and is not suitable for serious decompensated cirrhosis patients, such as multi-organ failure, abnormal coagulation function, etc. Besides, it should be mentioned that our study did not distinguish between bare and covered stents, which may underestimate the effectiveness of TIPS.

EIS has been supplanted by EVL as the main therapeutic strategy because of the growing evidence that EVL has lower complication rates (51, 64). EIS is associated with severe complications such as transient dysphagia, retrosternal chest discomfort, low-grade fever and esophageal ulceration. Complications occur in up to 40% of patients. ETA employs N-butyl-cyanoacrylate, a strong tissue adhesive used for hemostasis that causes endothelial fibrosis and venous obturation. ETA is associated with a rebleeding risk of 20–25% when endoscopic tissue adhesion achieves hemostasis (65). Our results showed that rebleeding was more frequent after ET than after TIPS or DSRS. However, patients had significantly lower rates of HE after ETA. According to the 2015 United Kingdom guidelines and the 2017 American Association for the Study of Liver Diseases guidelines, combined therapies are favored over EIS, EVL or ETA alone (66, 67). Similarly, based on our network meta-analysis, EVL + medication resulted in a higher 1-year OS rate compared with EVL, EIS or ETA alone and a lower treatment failure rate compared with EVL or ETA alone. EVL + EIS was superior to EVL or EIS alone in terms of bleeding-related mortality.

β-blockers such as propranolol and nadolol have been used for more than 30 years (5). Terlipressin and somatostatin are potent splanchnic vasoconstrictors that also have systemic circulatory effects; they increase arterial pressure and systemic vascular resistance, inhibit glucagon and other vasoactive peptides, and facilitate adrenergic vasoconstriction (68, 69). Our meta-analysis showed that simple conservative treatment offers little benefit. β-blockers, terlipressin and somatostatin drugs do not offer greater benefits compared with endoscopy or interventional therapy (29). However, Patch et al. (70) and Saran et al. (46) reported that propranolol is as effective as EVL in preventing variceal rebleeding within a median follow-up period of 1–2 years. This discrepancy may be attributed to the relatively small sample size in these studies; there is a clear difference in efficacy between medications alone and ET + medication (12, 45).

To the best of our knowledge, this is the first study to comprehensively analyze the safety and efficacy of various treatments for patients with portal hypertension and cirrhosis in terms of bleeding-related complications. Our study has several advantages. First, the data were extracted from 40 high-quality randomized controlled trials that involved over 4,000 patients in 13 countries. Second, multiple endpoints were observed, including 1-, 2-, and 3-year rebleeding rates, treatment failure, bleeding-related mortality, 1-, 2-, and 3-year OS rates, and HE.

Our study also had several limitations. First, some of the included subgroups were too small to evaluate effectively. Thus, several subgroup analyses (e.g., balloon-occluded retrograde transvenous obliteration, EVL + EIS + β-blocker, bare stent, covered stents etc.) were not performed. Second, patient characteristics that may have resulted in unavoidable methodological heterogeneity, such as Child–Pugh class, age and sex, varied among individual studies and could not be further addressed by subgroup or sensitivity analyses. Third, many different medications were used, including isosorbide mononitrate, somatostatin, octreotide, terlipressin and β-blockers, and there may have been differences in dosage among the studies.

According to our meta-analysis, TIPS and DSRS were superior to other therapies in terms of short-term and long-term bleeding control. However, these therapies may increase the risk of HE. There was no significant difference among the groups in the 1- or 3-year OS rate. Based on the complexity of the network meta-analysis model, the results of the meta-analysis are closely related to the model parameters, including the initial values and number of iterations. Therefore, the results of this meta-analysis should be interpreted with caution. Prospective RCTs are required to provide more data on TIPS, balloon-occluded retrograde transvenous obliteration and combination therapy.

## Conclusions

In conclusion, TIPS and DSRS should be given priority in patients with portal hypertension and cirrhosis to control rebleeding, which may not improve survival. ET together with medication may improve survival. Furthermore, medications should be used in combination with ET or other treatments rather than as the sole therapeutic intervention.

## Data Availability Statement

The original contributions presented in the study are included in the article/Supplementary Material, further inquiries can be directed to the corresponding author/s.

## Author Contributions

QY: conception and design and data collection. QY, WC, CY, and JY: data analysis, interpretation, and drafting the manuscript. TJ and HC: reviewed data analysis, interpretation, writing the manuscript, read, and approved the final manuscript. All authors contributed to the article and approved the submitted version.

## Conflict of Interest

The authors declare that the research was conducted in the absence of any commercial or financial relationships that could be construed as a potential conflict of interest.

## Publisher's Note

All claims expressed in this article are solely those of the authors and do not necessarily represent those of their affiliated organizations, or those of the publisher, the editors and the reviewers. Any product that may be evaluated in this article, or claim that may be made by its manufacturer, is not guaranteed or endorsed by the publisher.

## Abbreviations

JAGS: Just Another Gibbs Sampler
MCMC: Markov Chain Monte Carlo chain
PSRF: Potential Scale Reduction Factor
CI: Confidence interval
OS: Overall survival
RR: Odds ratio
RCT: Randomized controlled trial
PRISMA-P: Preferred Reporting Items for Systematic Review and Meta-Analysis Protocols
EIS: endoscopic sclerotherapy
EVL: endoscopic variceal ligation
ETA: endoscopic tissue adhesion
ISMN: isosorbide mononitrate
TIPS: transjugular intrahepatic portosystemic shunt
DSRS: distal splenorenal shunt.
