# Supplementary material for: Efficacy and Safety of Treatments for Patients With Portal Hypertension and Cirrhosis: A Systematic Review and Bayesian Network Meta-Analysis
Source: Front Med (Lausanne). 2021 Sep 3;8:712918. doi: 10.3389/fmed.2021.712918 (PMC8446274; doi:10.3389/fmed.2021.712918)
Supplement: Supplementary file 1 [file Data_Sheet_1.DOCX]

**Supplementary data**

**Efficacy and safety of treatments for patients with portal hypertension and cirrhosis: a systematic review and Bayesian network meta-analysis**

Qigu Yao^1,2^ **^†^**, Wenyi Chen^1,2^**^†^**, Cuilin Yan^1^, Jong Yu^1,2^, Tianan Jiang^2,3^, Hongcui Cao^1,2,4^*

1 State Key Laboratory for Diagnosis and Treatment of Infectious Diseases, The First Affiliated Hospital, Zhejiang University School of Medicine, 79 Qingchun Rd., Hangzhou City 310003, China

2 National Clinical Research Center for Infectious Diseases, 79 Qingchun Rd., Hangzhou City 310003, China

3 Department of Ultrasound, The First Affiliated Hospital, Zhejiang University School of Medicine, 79 Qingchun Rd., Hangzhou City 310003, China

4 Zhejiang Provincial Key Laboratory for Diagnosis and Treatment of Aging and Physic-chemical Injury Diseases, 79 Qingchun Rd, Hangzhou 310003, China

**^†^** † These authors contributed equally.

*Corresponding author:

Hongcui Cao, State Key Laboratory for Diagnosis and Treatment of Infectious Diseases, The First Affiliated Hospital, Zhejiang University School of Medicine, 79 Qingchun Rd., Hangzhou City 310003, China

Tel: 86-571-87236451; Fax: 86-571-87236459

E-mail: [hccao@zju.edu.cn](mailto:hccao@zju.edu.cn)

**Supplementary Figure 1.** Trace plot and density diagram of rebleeding all sources based on different pairwise comparisons. In trace plots, the fluctuation of Markov Chain Monte Carlo chain could not be recognized by the naked eye. In the density diagram, the bandwidth tended to be zero and stable, and a smooth curve that conformed to the normal distribution. Two kinds of plot all indicated that the model had good convergence. EVL, endoscopic variceal ligation; TIPS, transjugular intrahepatic portosystemic shunt; DSRS, distal splenorenal shunt; EIS, endoscopic injection sclerotherapy; ETA, endoscopic tissue.


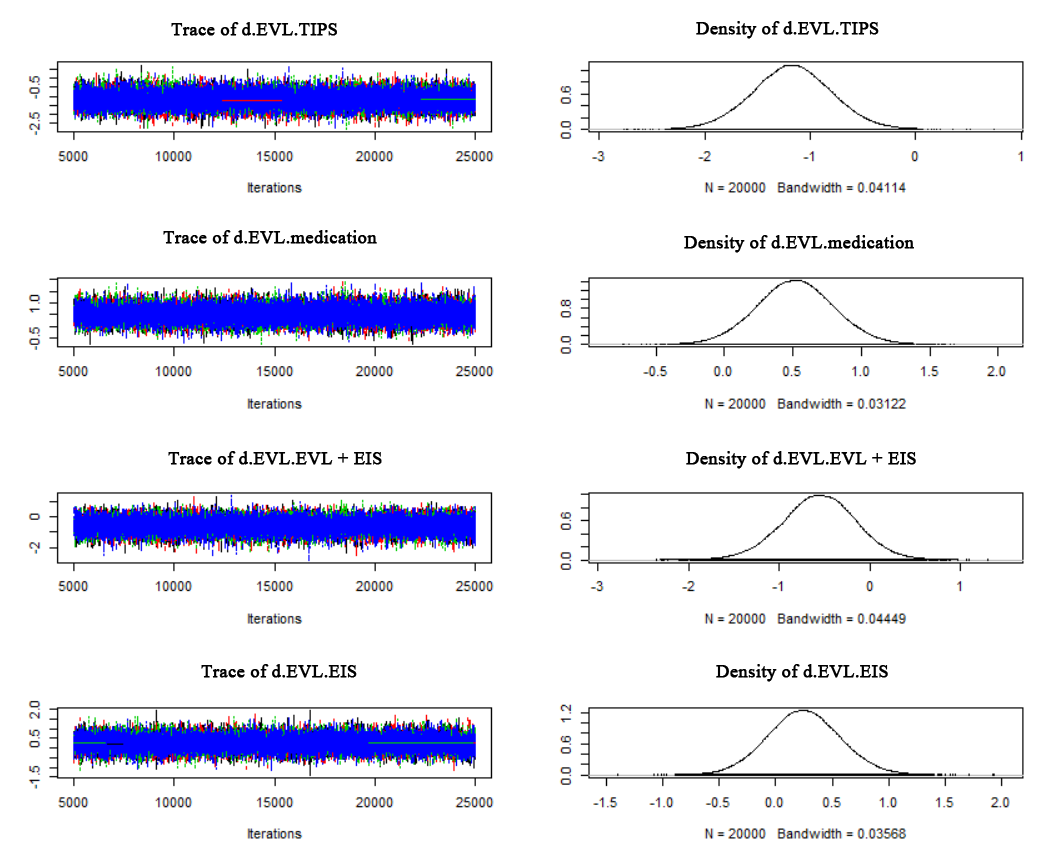


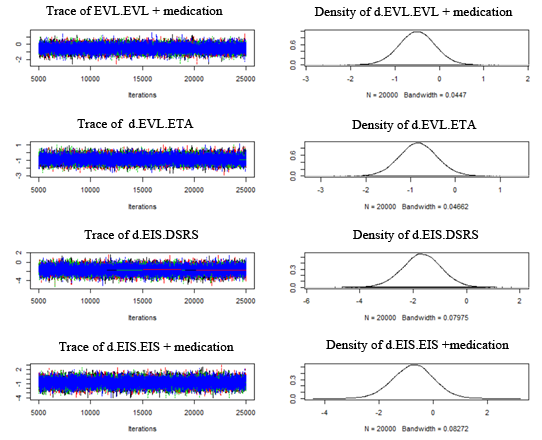


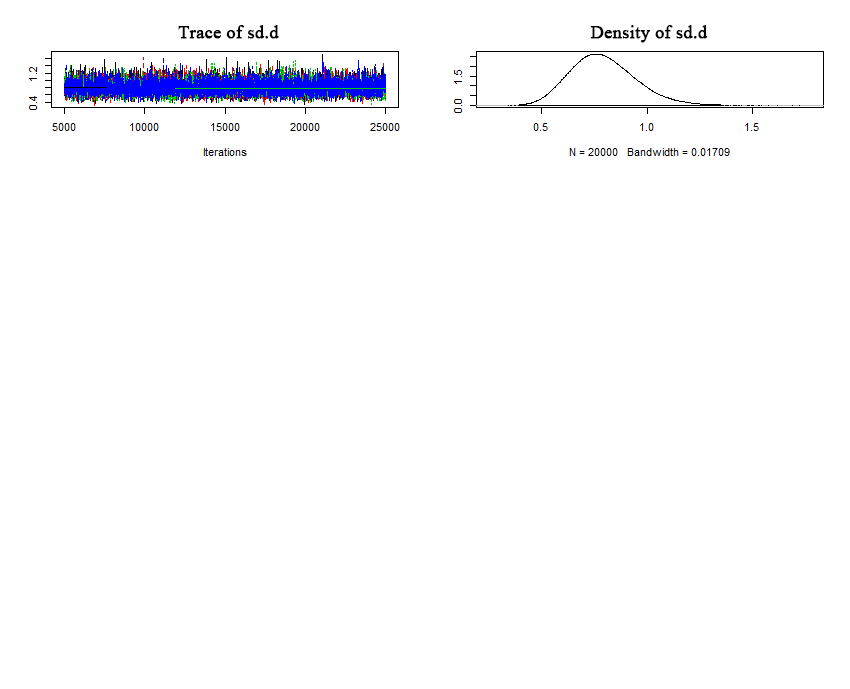


**Supplementary Figure 2.** The plot of Brooks-Gelman-Rubin diagnostic of rebleeding all sources based on different pairwise comparisons. The potential scale reduction factor for each analysis was close to 1.0, which the degree of convergence was considered satisfactory. EVL, endoscopic variceal ligation; TIPS, transjugular intrahepatic portosystemic shunt; DSRS, distal splenorenal shunt; EIS, endoscopic injection sclerotherapy; ETA, endoscopic tissue.


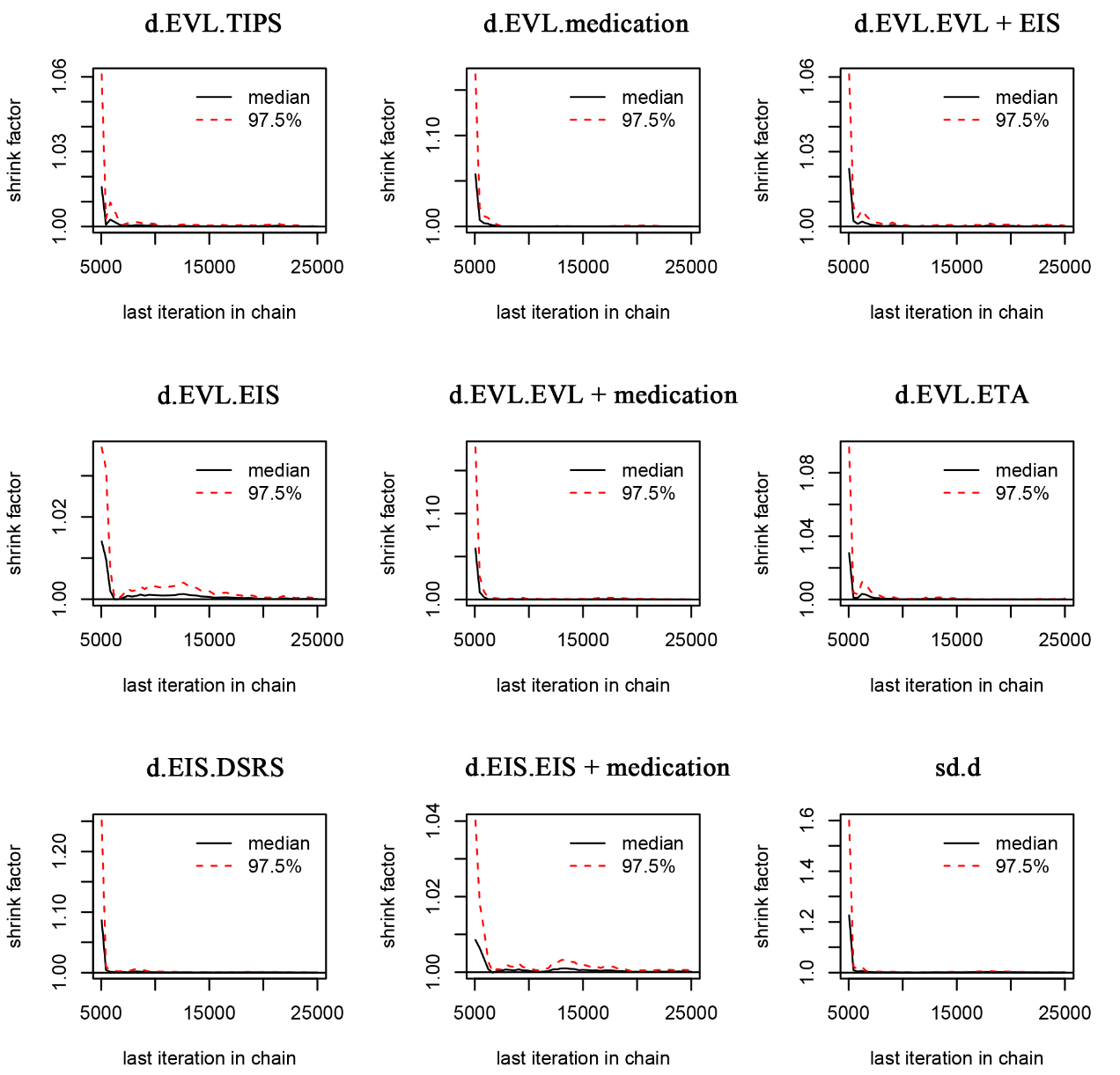


**Supplementary Figure 3.** Forest plot of odds ratios of 1-year rebleeding based on different pairwise comparisons. EVL, endoscopic variceal ligation; TIPS, transjugular intrahepatic portosystemic shunt; ETA, endoscopic tissue.


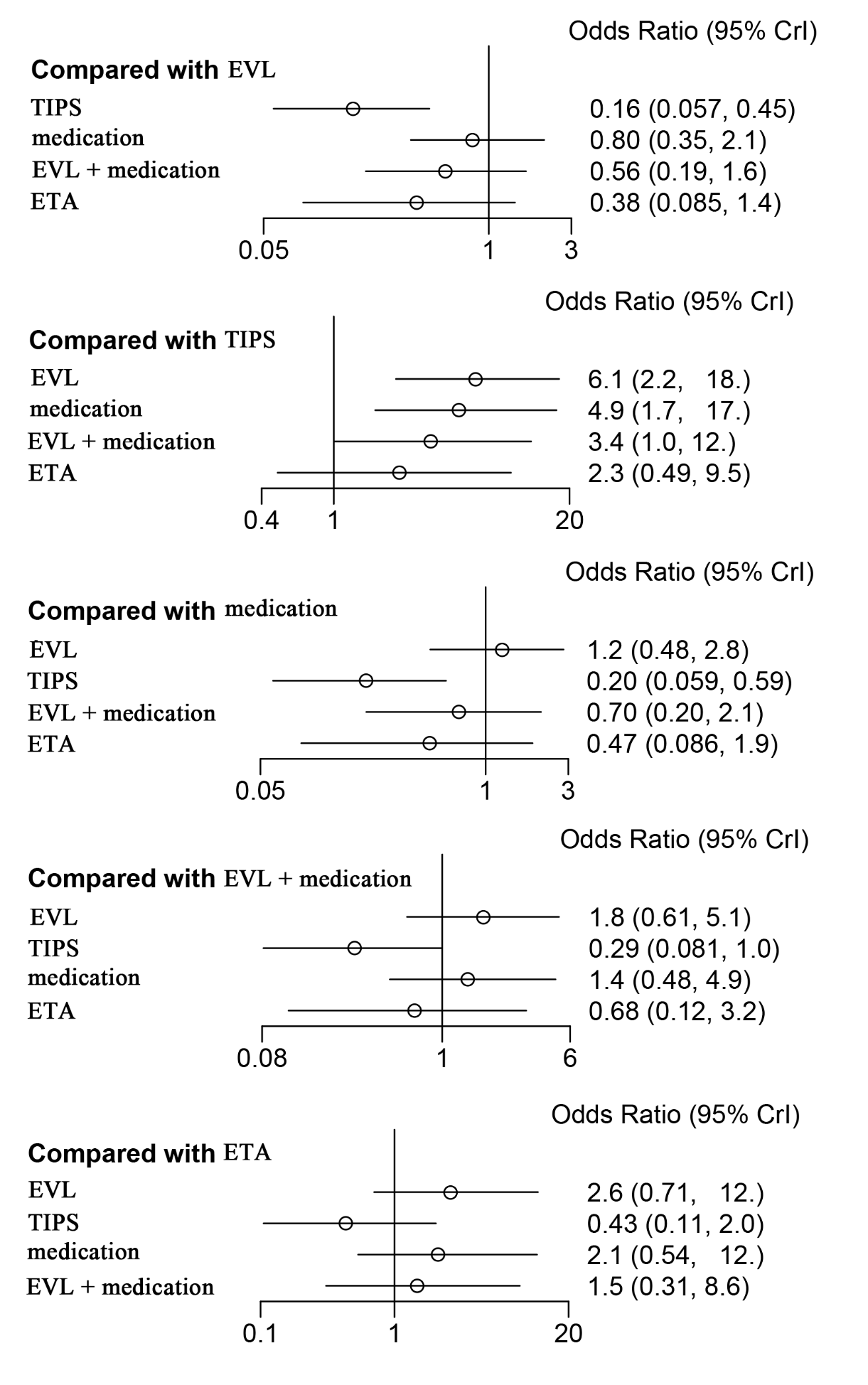


**Supplementary Figure 4.** Forest plot of odds ratios of 2-year rebleeding based on different pairwise comparisons. EVL, endoscopic variceal ligation; TIPS, transjugular intrahepatic portosystemic shunt; ETA, endoscopic tissue.


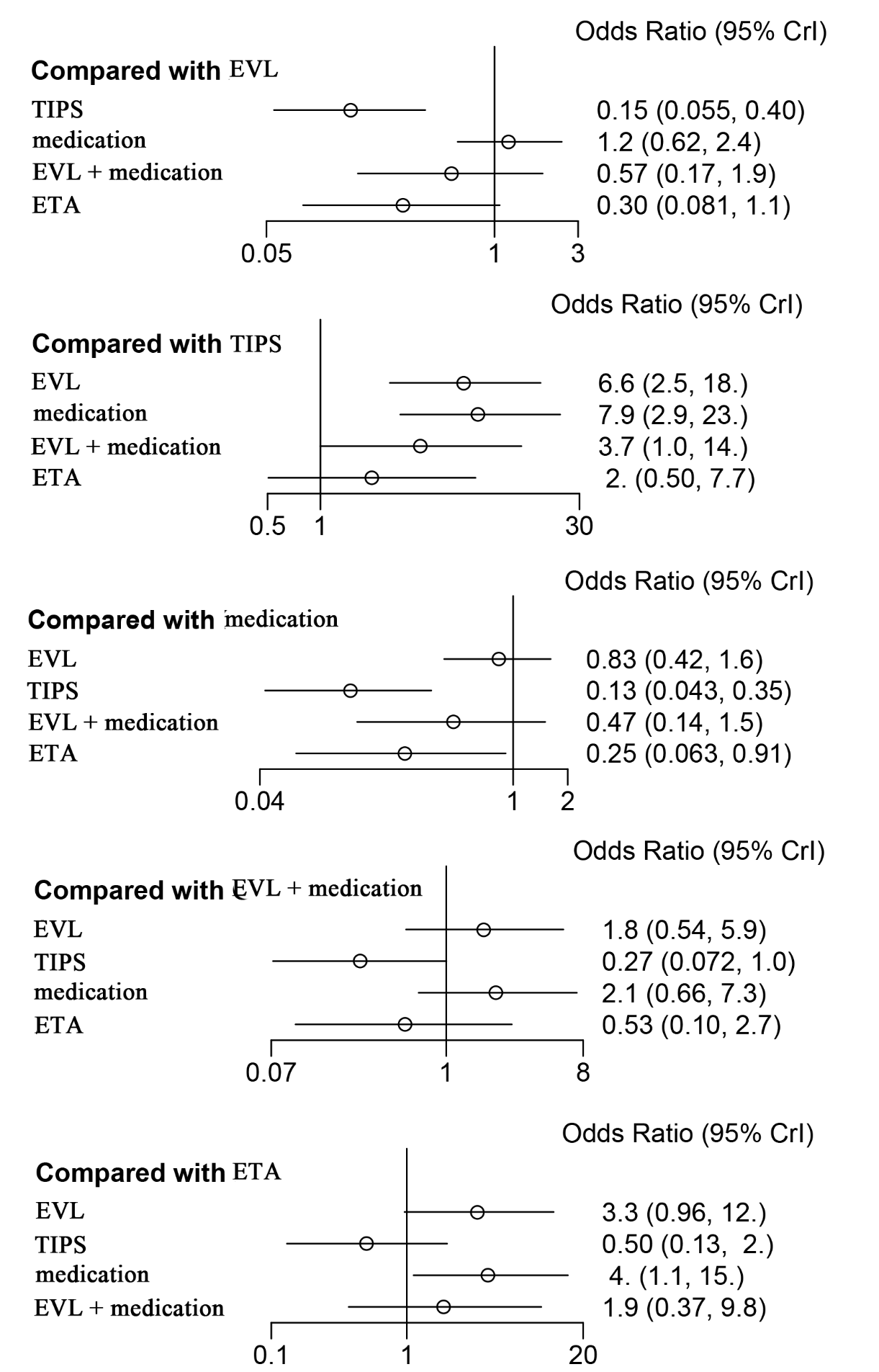


**Supplementary Figure 5.** Forest plot of odds ratios of 3-year rebleeding based on different pairwise comparisons. EVL, endoscopic variceal ligation; TIPS, transjugular intrahepatic portosystemic shunt; ETA, endoscopic tissue.


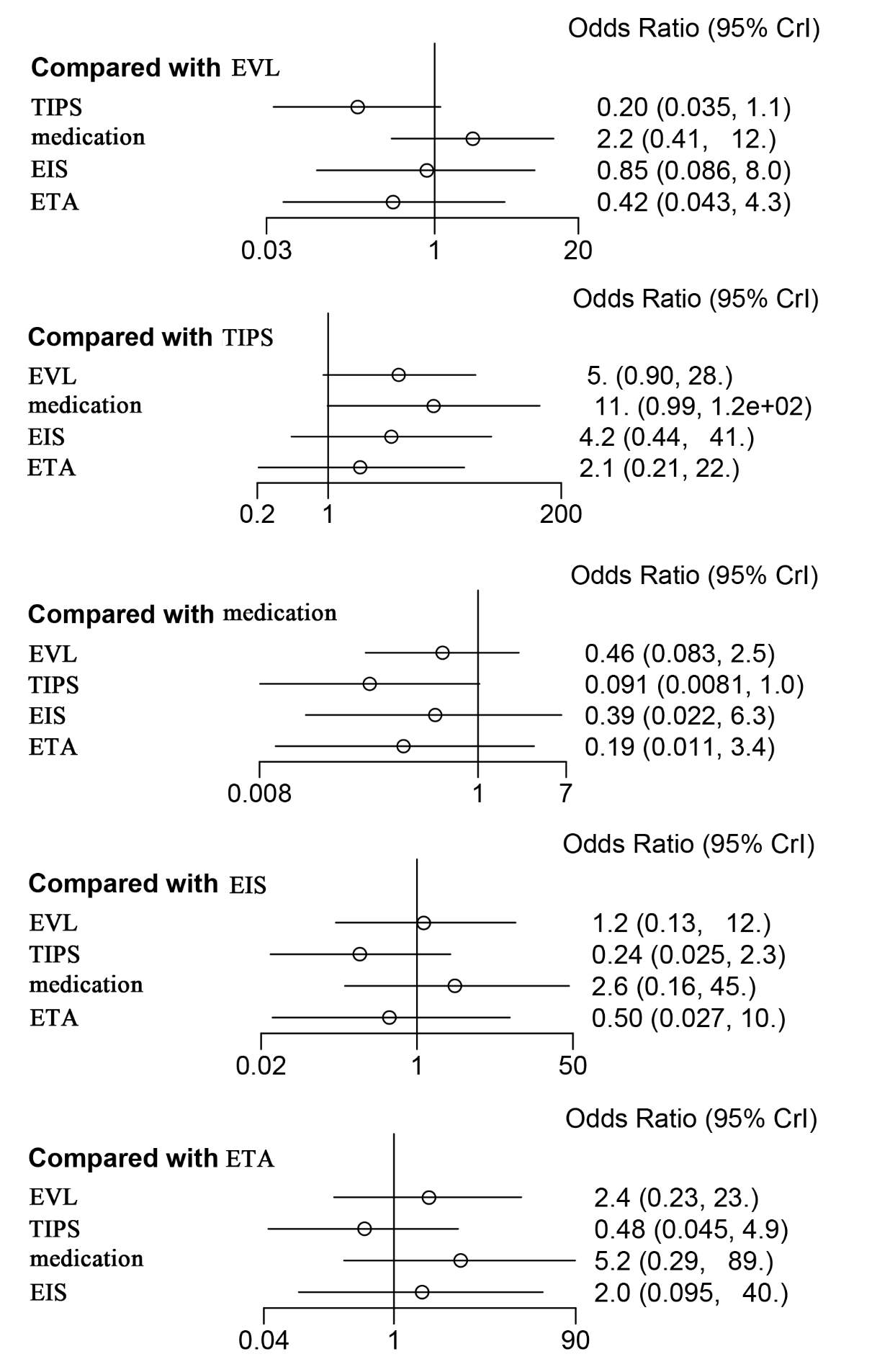


**Supplementary Figure 6.** Ranking of 1-, 2- or 3-year rebleeding among different therapies. EVL, endoscopic variceal ligation; TIPS, transjugular intrahepatic portosystemic shunt; ETA, endoscopic tissue.


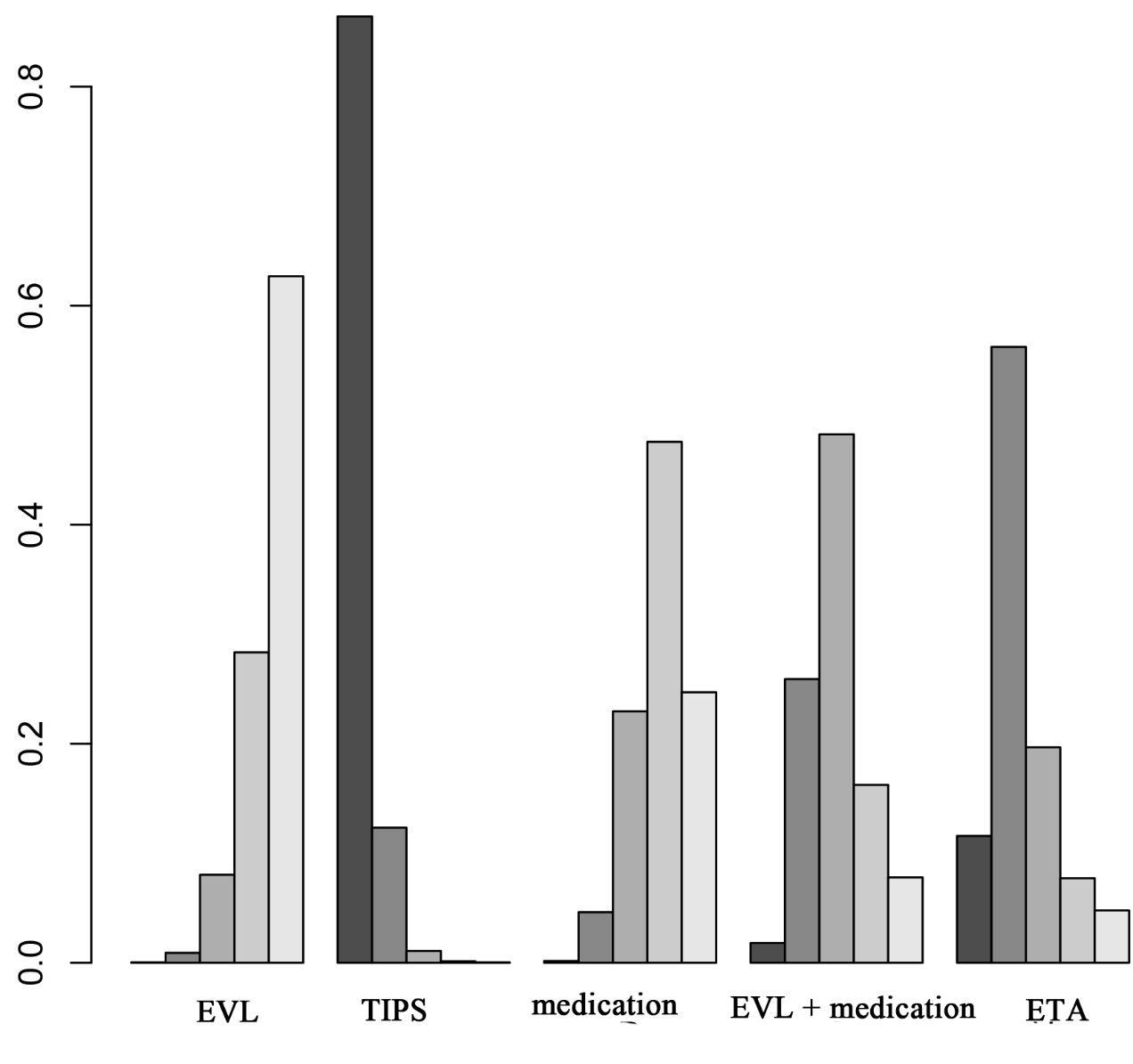


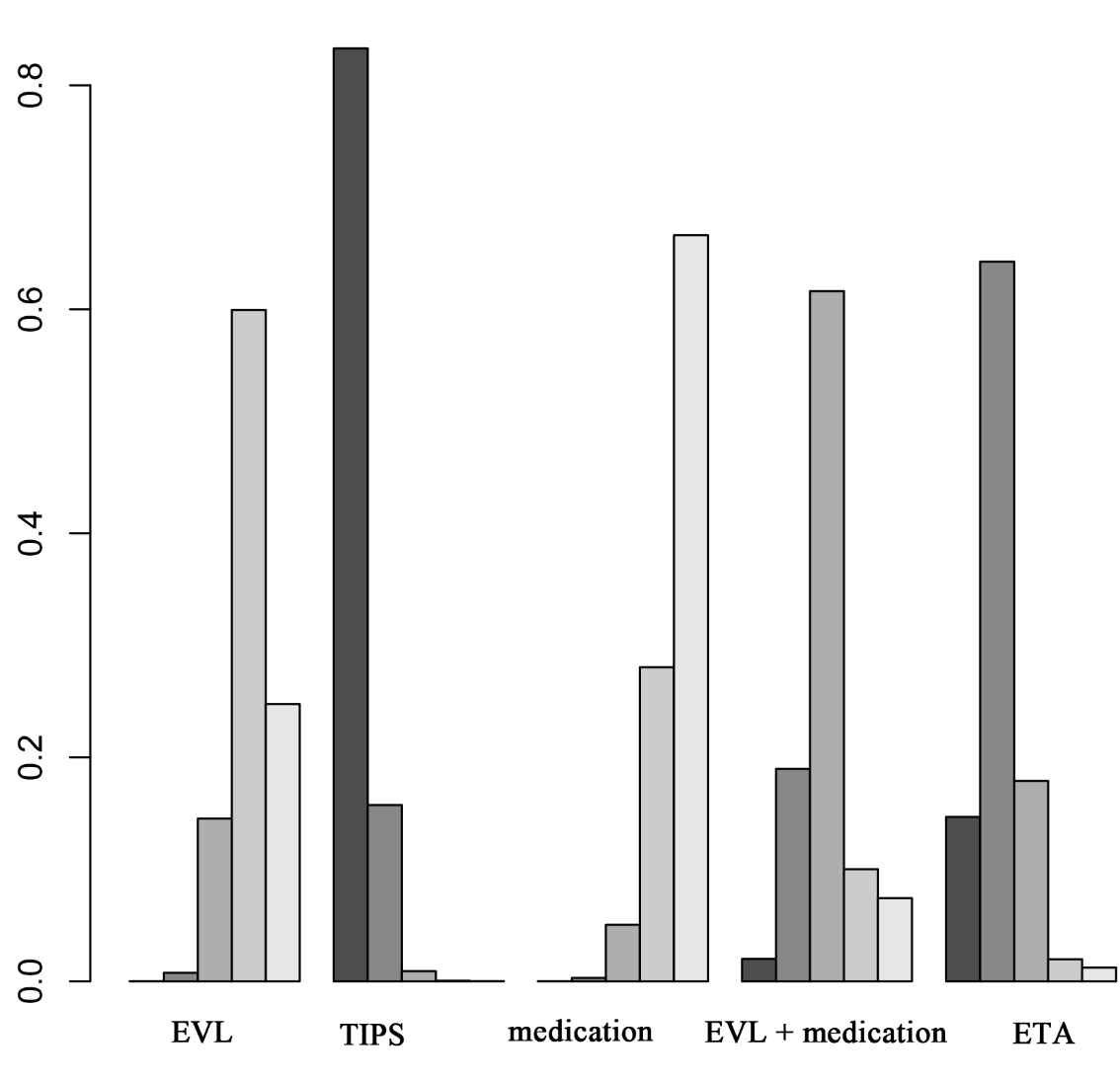


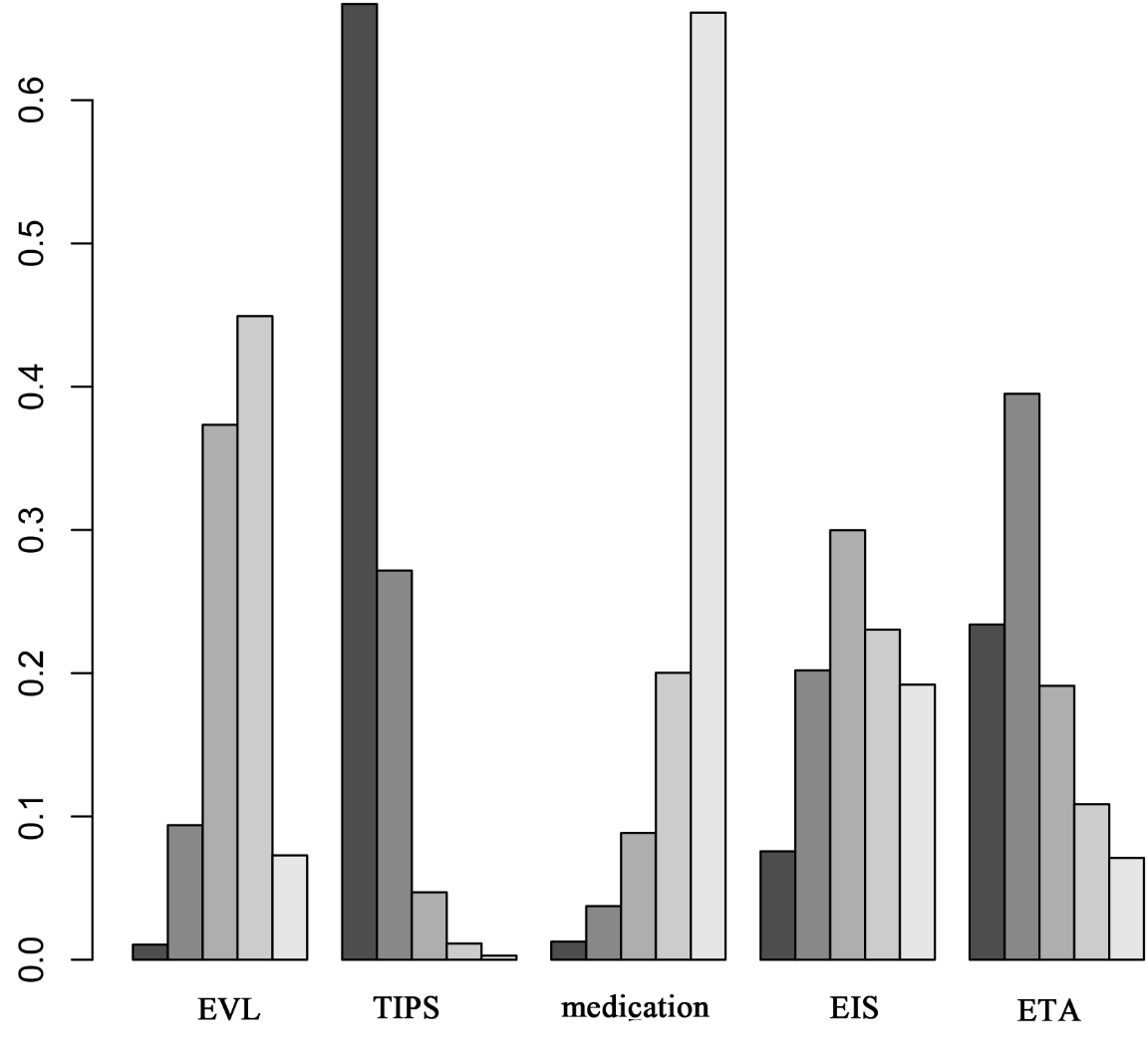


**Supplementary Figure 7.** Forest plot of odds ratios of bleeding-related mortality based on different pairwise comparisons. EVL, endoscopic variceal ligation; TIPS, transjugular intrahepatic portosystemic shunt; EIS, endoscopic injection sclerotherapy; ETA, endoscopic tissue.


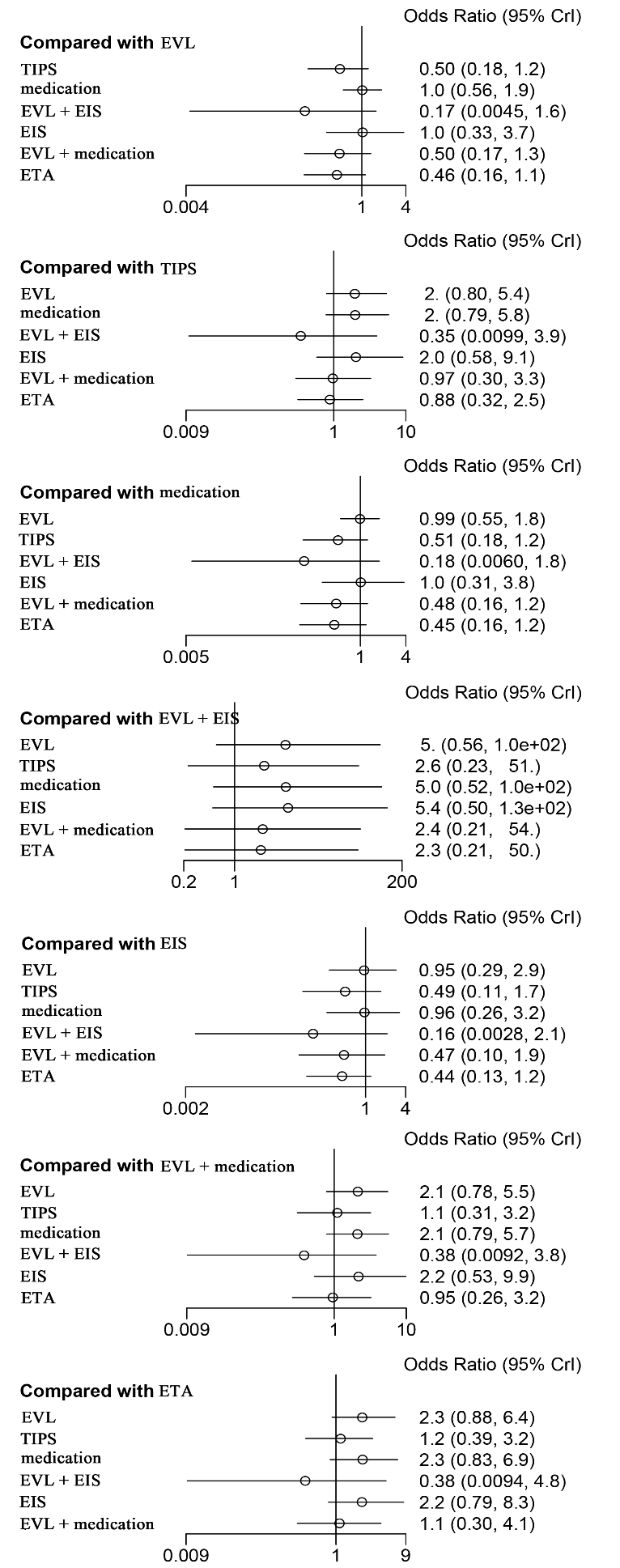


**Supplementary Figure 8.** Ranking of bleeding-related mortality among different therapies. EVL, endoscopic variceal ligation; TIPS, transjugular intrahepatic portosystemic shunt; EIS, endoscopic injection sclerotherapy; ETA, endoscopic tissue.


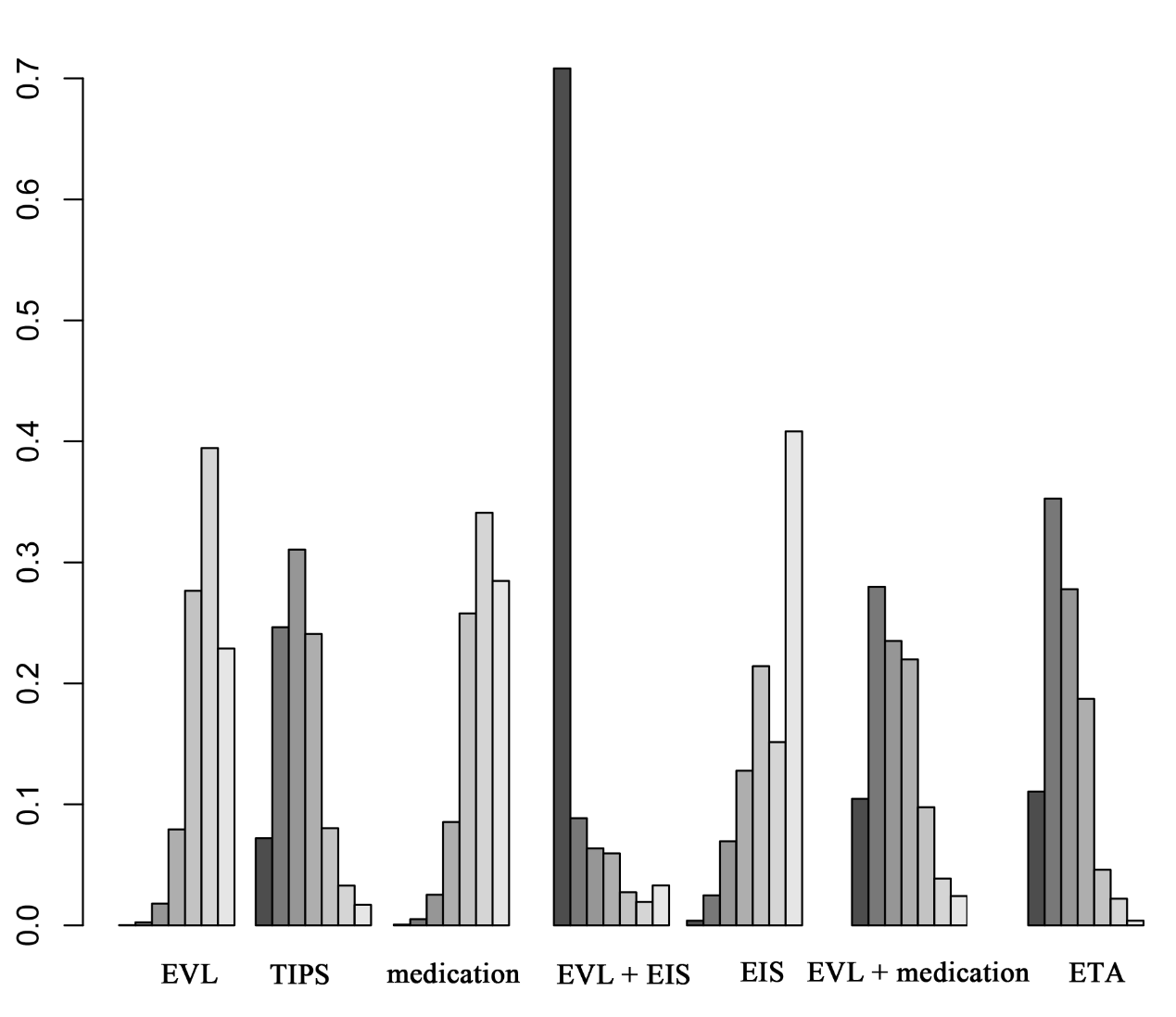


**Supplementary Figure 9.** Forest plot of the odds ratios for 1-year OS based on different pairwise comparisons. EVL, endoscopic variceal ligation; TIPS, transjugular intrahepatic portosystemic shunt; DSRS, distal splenorenal shunt; EIS, endoscopic injection sclerotherapy; ETA, endoscopic tissue.


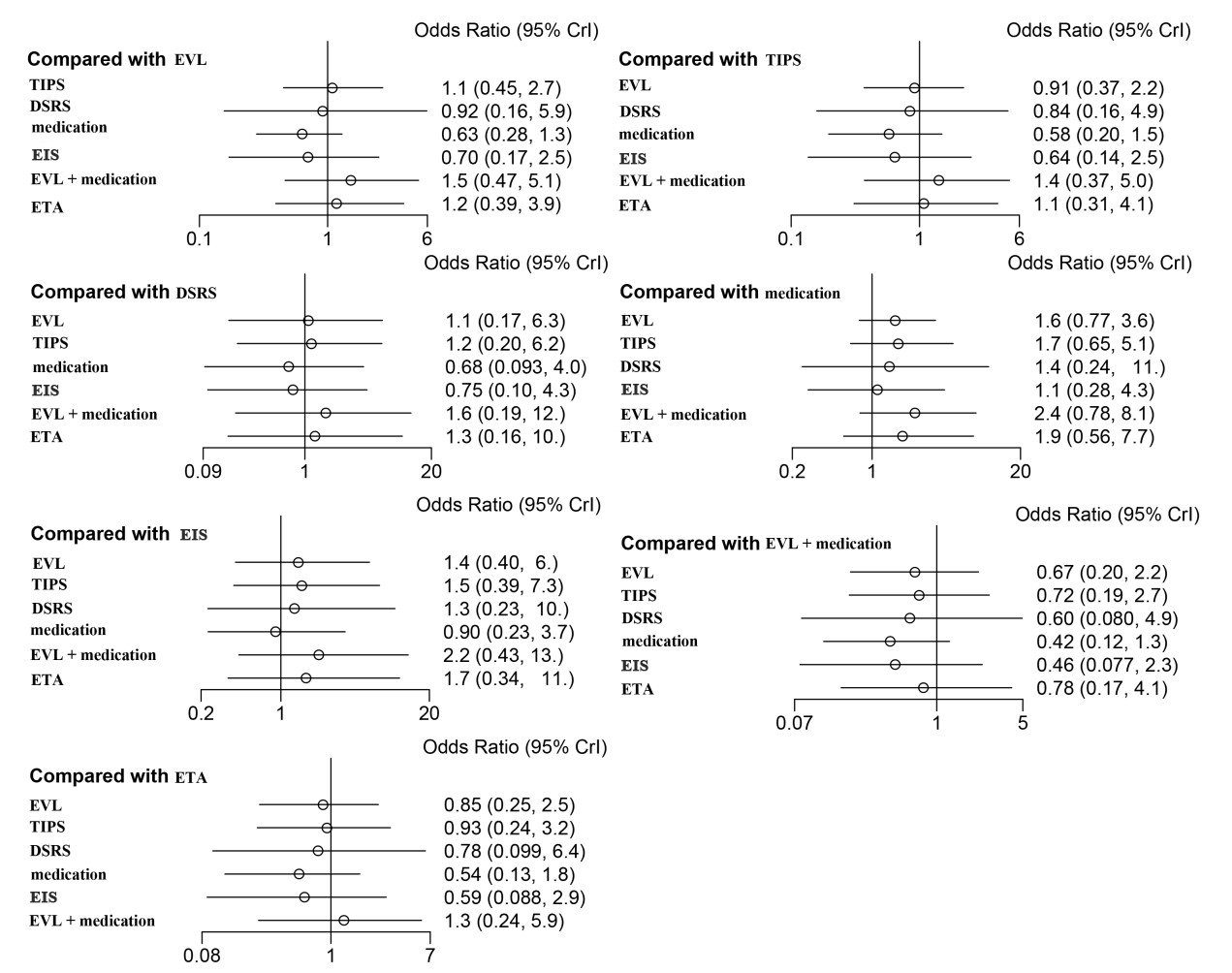


**Supplementary Figure 10.** Forest plot of odds ratios of 2-year OS based on different pairwise comparisons. EVL, endoscopic variceal ligation; TIPS, transjugular intrahepatic portosystemic shunt; DSRS, distal splenorenal shunt; EIS, endoscopic injection sclerotherapy; ETA, endoscopic tissue.


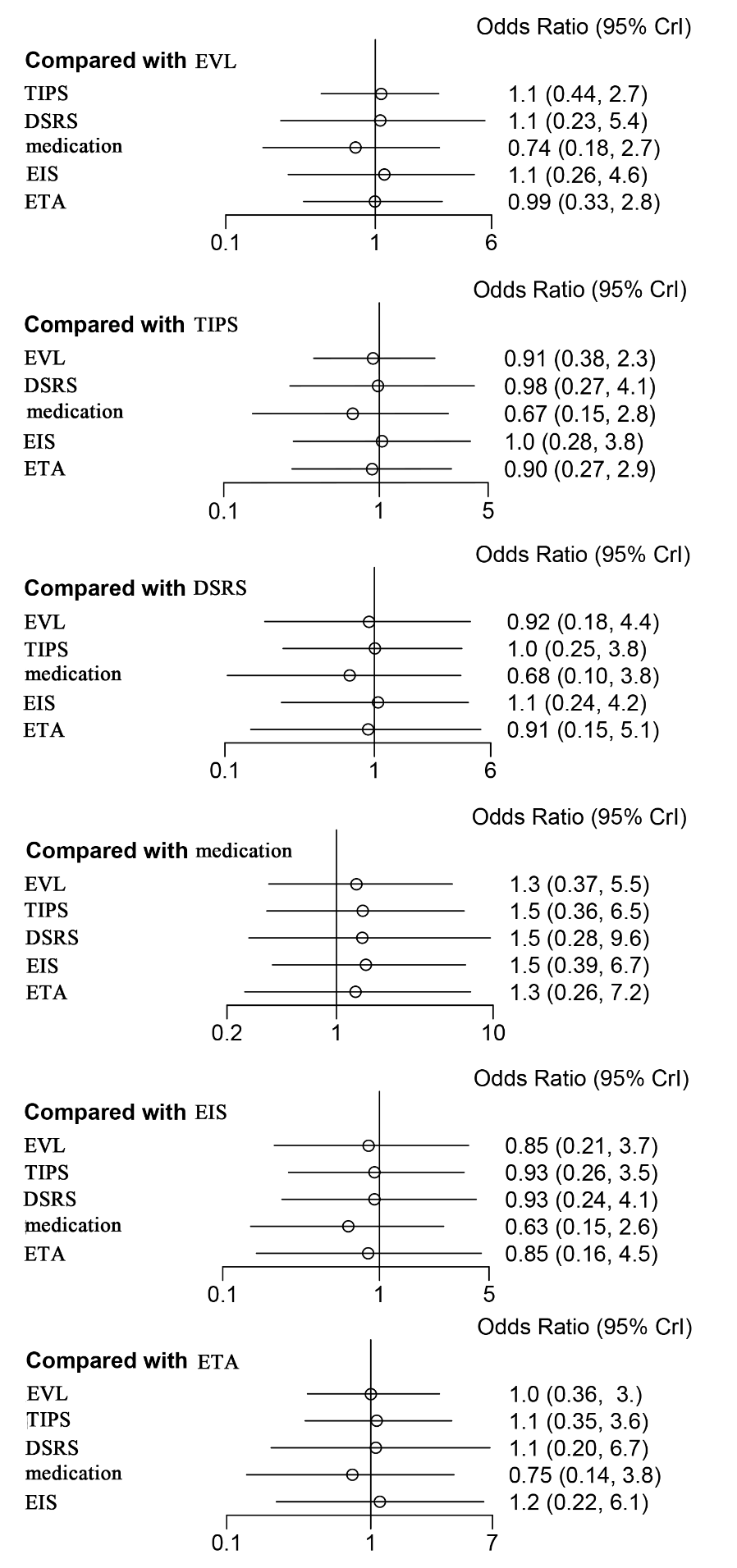


**Supplementary Figure 11.** Forest plot of odds ratios of 3-year OS based on different pairwise comparisons. EVL, endoscopic variceal ligation; TIPS, transjugular intrahepatic portosystemic shunt; DSRS, distal splenorenal shunt; EIS, endoscopic injection sclerotherapy; ETA, endoscopic tissue.


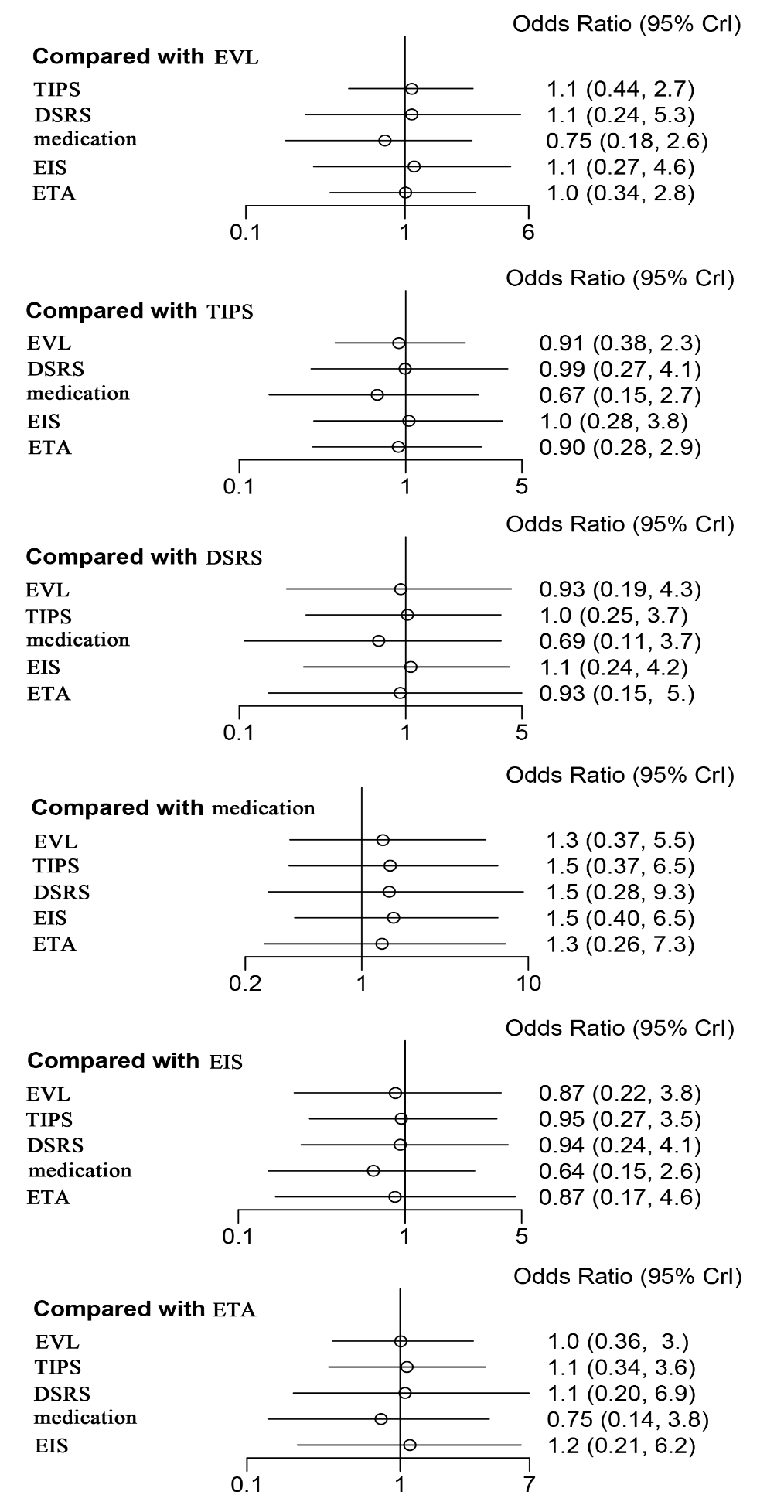


**Supplementary Figure 12.** Ranking of 1-year OS among the different therapies. EVL, endoscopic variceal ligation; TIPS, transjugular intrahepatic portosystemic shunt; DSRS, distal splenorenal shunt; EIS, endoscopic injection sclerotherapy; ETA, endoscopic tissue.

**
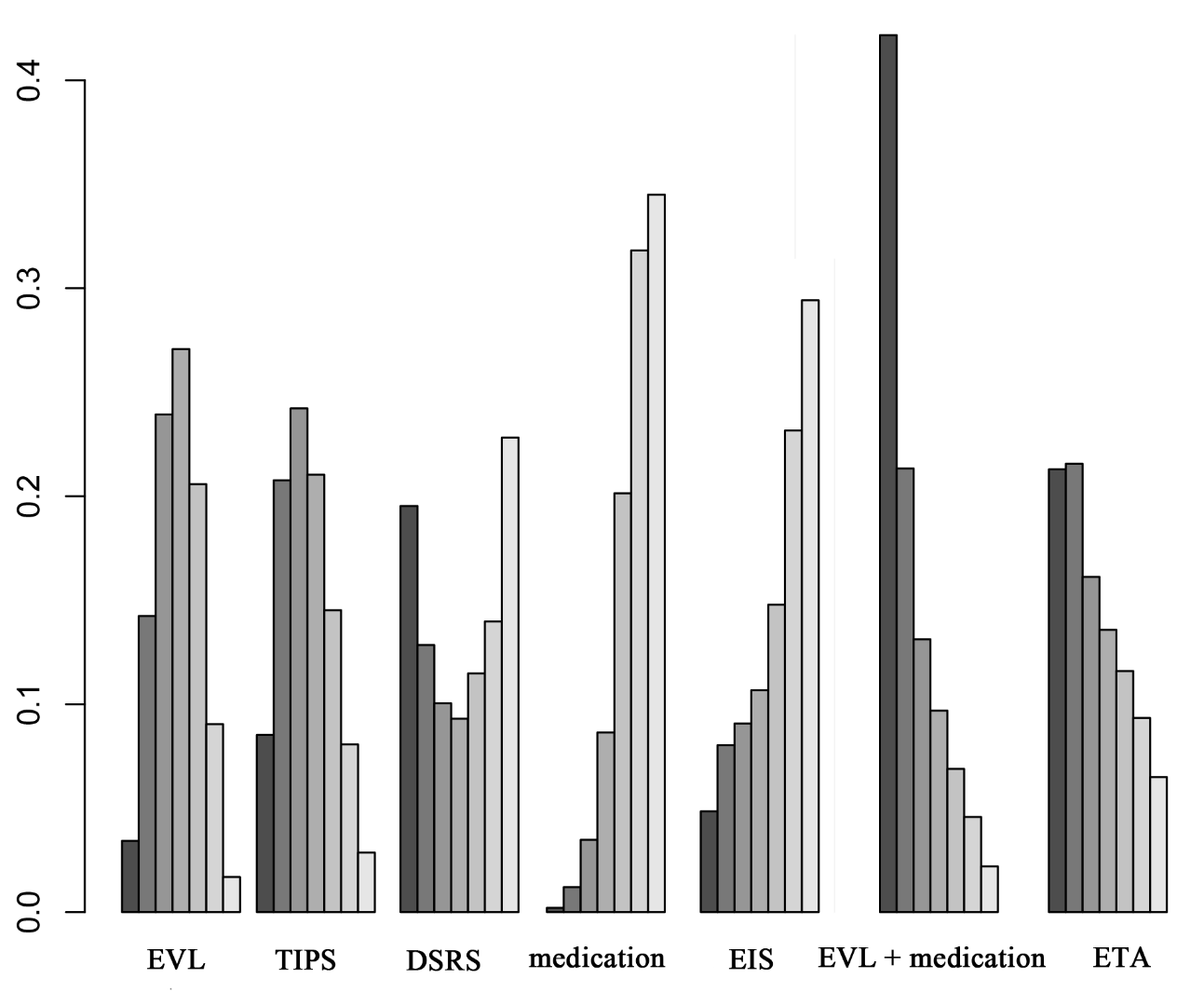
**

**Supplementary Figure 13.** Ranking of 2-year OS among different therapies. EVL, endoscopic variceal ligation; TIPS, transjugular intrahepatic portosystemic shunt; DSRS, distal splenorenal shunt; EIS, endoscopic injection sclerotherapy; ETA, endoscopic tissue.

**
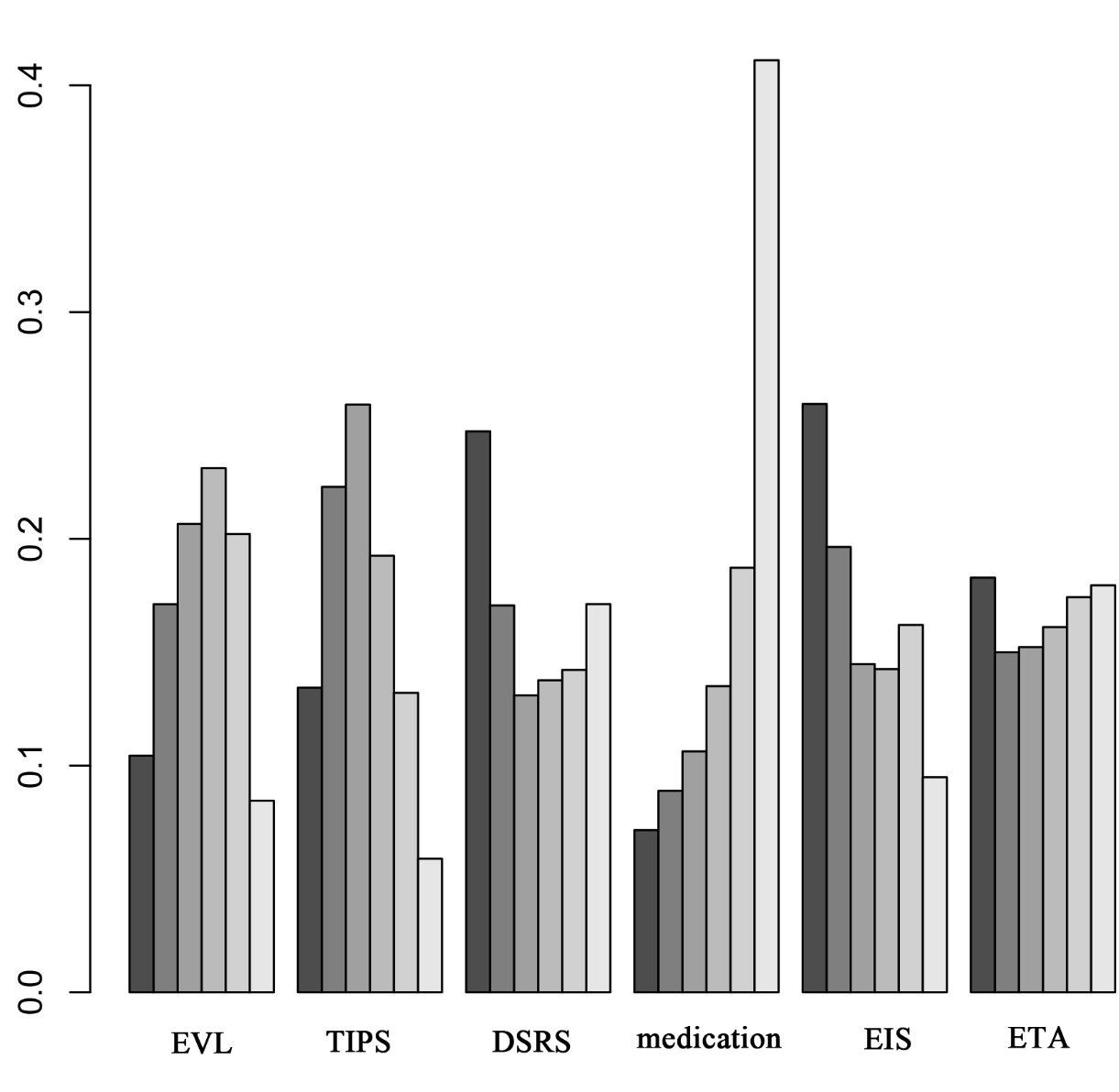
**

**Supplementary Figure 14.** Ranking of 3-year OS among different therapies. EVL, endoscopic variceal ligation; TIPS, transjugular intrahepatic portosystemic shunt; DSRS, distal splenorenal shunt; EIS, endoscopic injection sclerotherapy; ETA, endoscopic tissue.


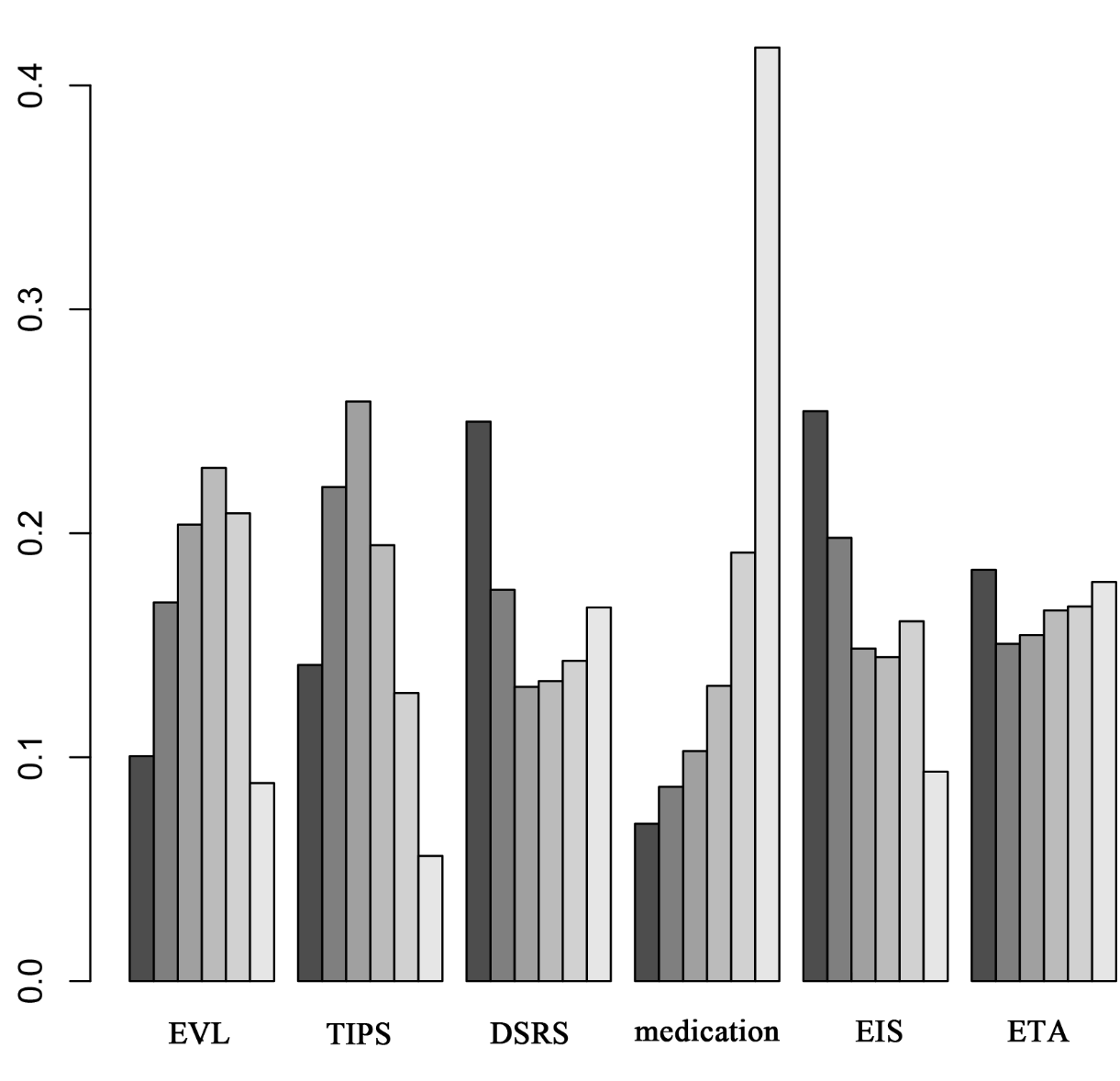


**Supplementary Figure 15.** Forest plot of odds ratios of treatment failure based on different pairwise comparisons. EVL, endoscopic variceal ligation; ETA, endoscopic tissue.


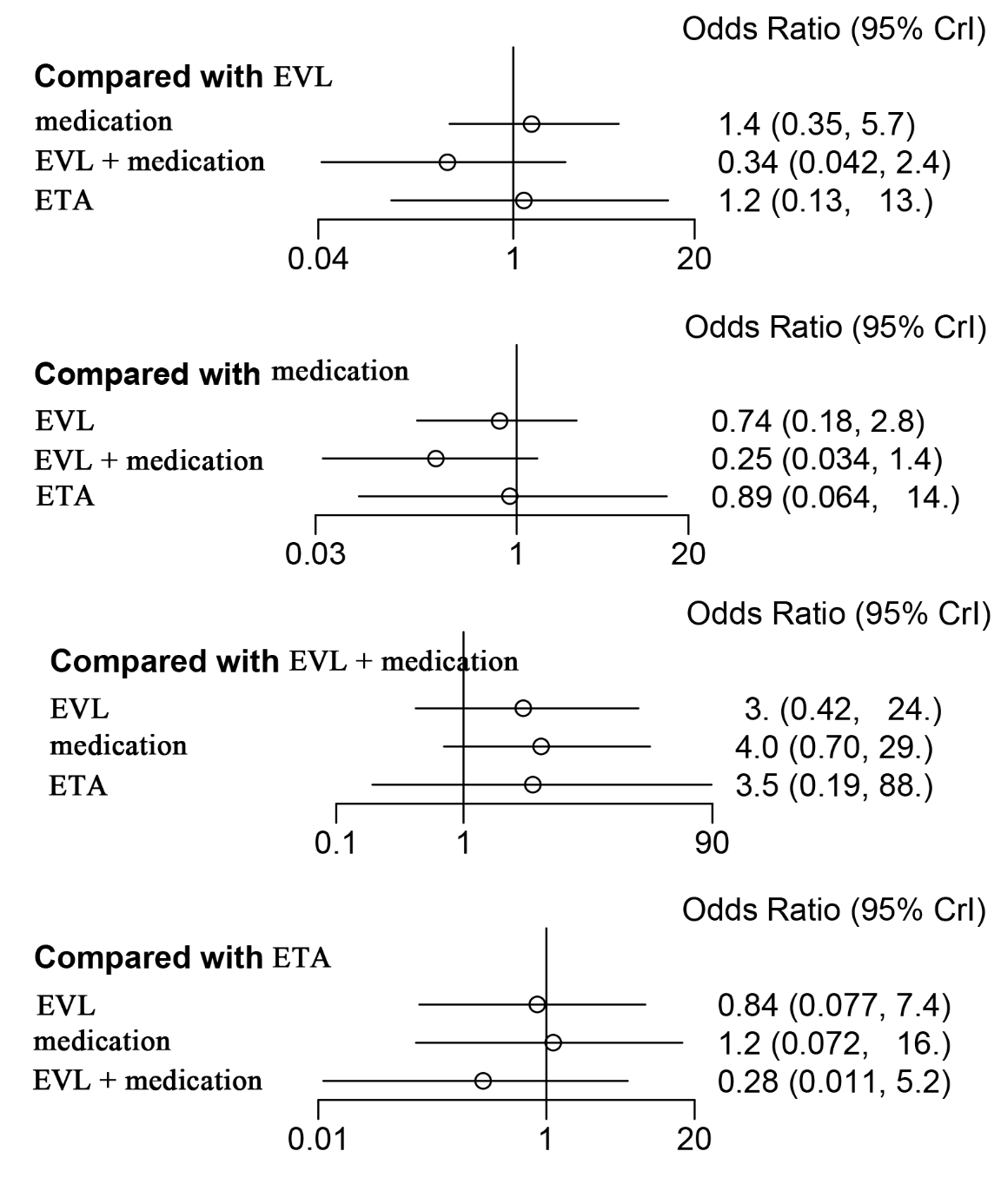


**Supplementary Figure 16.** Ranking of treatment failure among different therapies. EVL, endoscopic variceal ligation; ETA, endoscopic tissue.


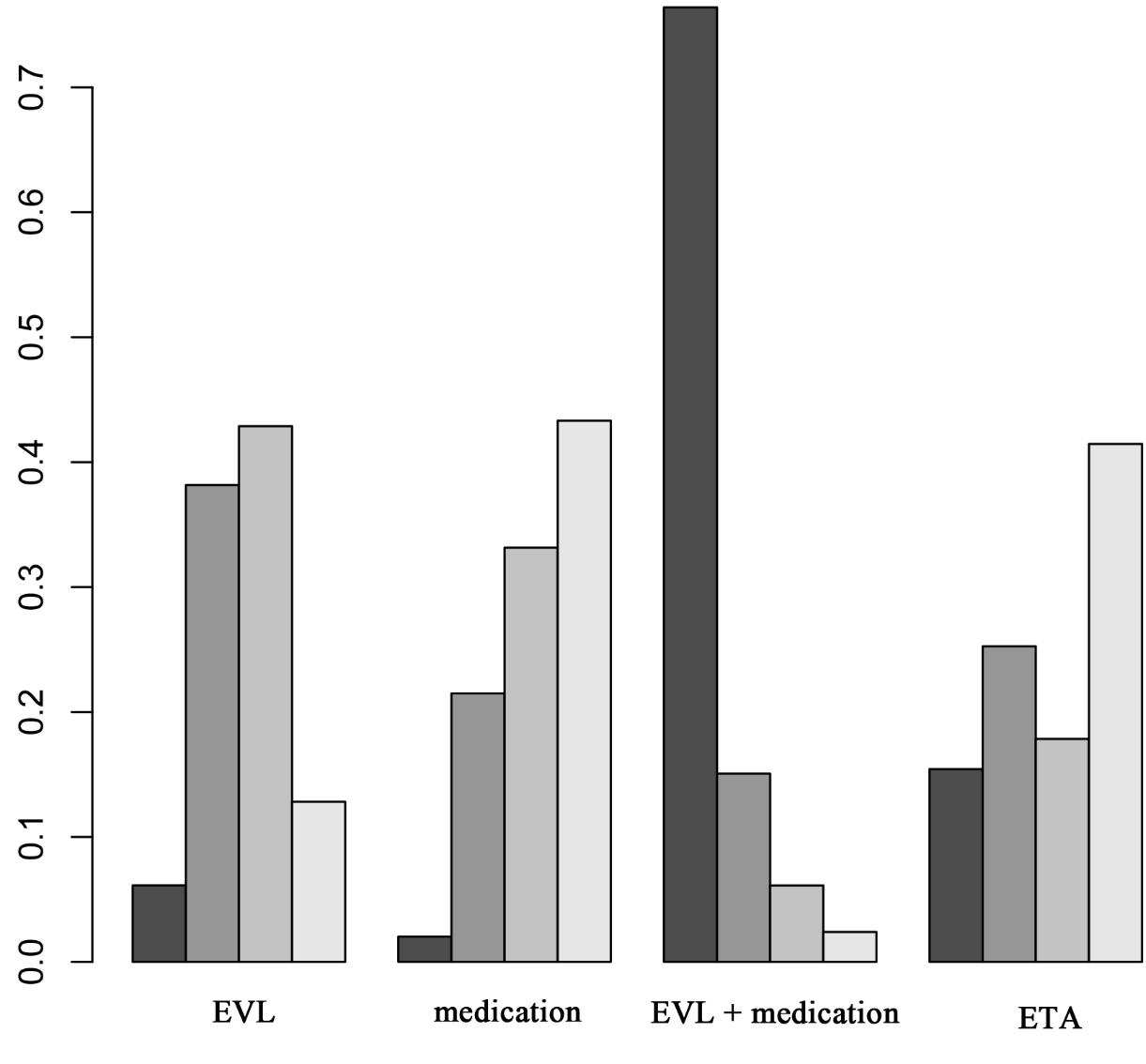


**Supplementary Figure 17.** Forest plot of the odds ratios for HE based on different pairwise comparisons. EVL, endoscopic variceal ligation; TIPS, transjugular intrahepatic portosystemic shunt; DSRS, distal splenorenal shunt; EIS, endoscopic injection sclerotherapy; ETA, endoscopic tissue.


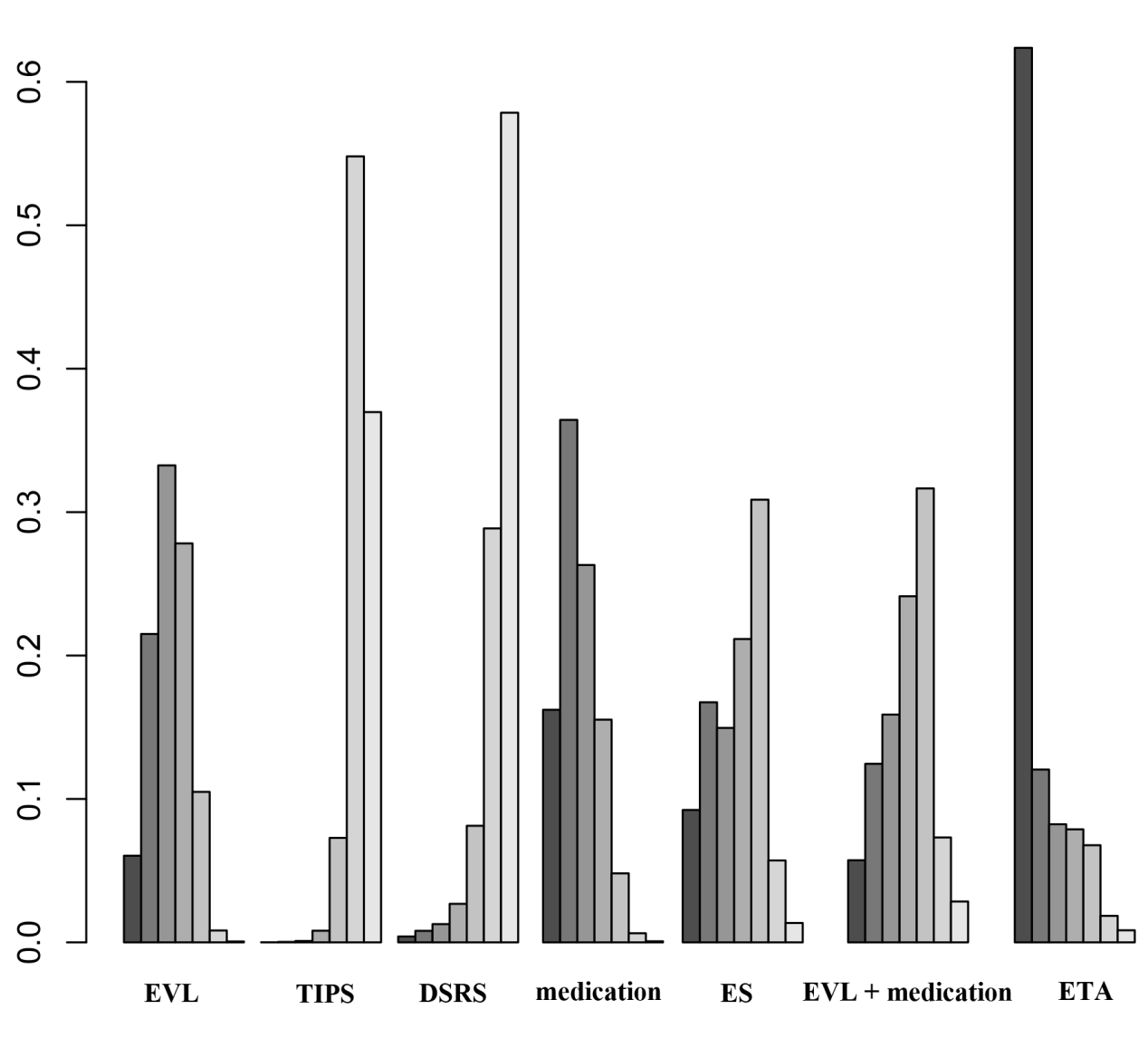


**Supplementary Figure 18.** Ranking of HE among different therapies. EVL, endoscopic variceal ligation; TIPS, transjugular intrahepatic portosystemic shunt; DSRS, distal splenorenal shunt; EIS, endoscopic injection sclerotherapy; ETA, endoscopic tissue.


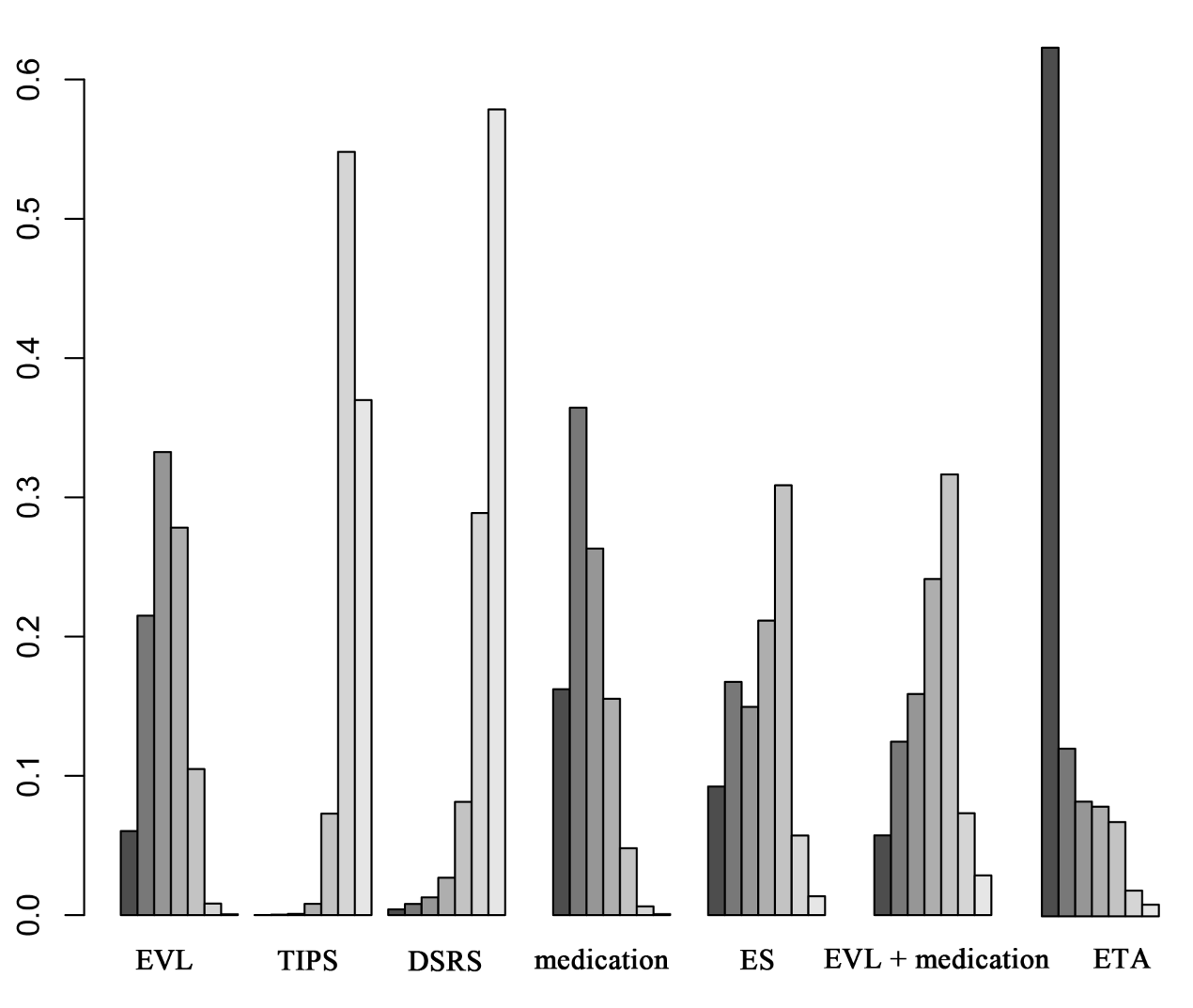


**Supplementary Table 1.** Quality assessment of studies included.

| **Author, year,**  **Study (RCT)** | **Sequence**  **Generation** | | **Allocation**  **Concealment** | | **Blinding** | **Incomplete**  **outcome data** | **Selective**  **outcome reporting** | **Free of**  **other bias** |
| --- | --- | --- | --- | --- | --- | --- | --- | --- |
| Argonz 2000 | | unclear risk | low risk | unclear risk | | low risk | low risk | low risk |
| Hou 2000 | | unclear risk | low risk | unclear risk | | low risk | low risk | unclear risk |
| Lo 2000 | | low risk | low risk | unclear risk | | low risk | unclear risk | low risk |
| Orozco 2000 | | low risk | low risk | unclear risk | | low risk | low risk | unclear risk |
| Villanueva 2001 | | low risk | low risk | low risk | | low risk | low risk | low risk |
| Pomier-Layrargues 2001 | | unclear risk | low risk | unclear risk | | low risk | low risk | low risk |
| Hou 2001 | | unclear risk | low risk | unclear risk | | low risk | low risk | unclear risk |
| Cheng 2001 | | low risk | low risk | unclear risk | | low risk | unclear risk | low risk |
| Narahara 2001 | | low risk | low risk | low risk | | low risk | low risk | low risk |
| Sauer 2002 | | unclear risk | low risk | low risk | | low risk | low risk | low risk |
| Gülberg 2002 | | low risk | low risk | unclear risk | | low risk | low risk | low risk |
| Escorsell 2002 | | unclear risk | low risk | unclear risk | | low risk | low risk | low risk |
| Viazis 2002 | | low risk | low risk | low risk | | low risk | low risk | unclear risk |
| Avgerinos 2004 | | low risk | low risk | unclear risk | | low risk | low risk | low risk |
| Schepke 2004 | | low risk | low risk | unclear risk | | low risk | low risk | low risk |
| Peña 2005 | | low risk | low risk | unclear risk | | low risk | low risk | low risk |
| Sarin 2005 | | low risk | low risk | unclear risk | | low risk | low risk | low risk |
| Shah 2005 | | low risk | low risk | low risk | | low risk | low risk | low risk |
| Zargar 2005 | | unclear risk | low risk | low risk | | low risk | low risk | unclear risk |
| Chen 2006 | | low risk | low risk | low risk | | low risk | low risk | low risk |
| Santambrogio 2006 | | low risk | low risk | unclear risk | | low risk | unclear risk | low risk |
| Romero 2006 | | low risk | low risk | low risk | | low risk | low risk | low risk |
| Henderson 2006 | | low risk | low risk | low risk | | low risk | low risk | low risk |
| Tan 2006 | | low risk | low risk | unclear risk | | low risk | low risk | low risk |
| Lo 2007 | | low risk | low risk | unclear risk | | low risk | low risk | low risk |
| Morales 2007 | | low risk | low risk | unclear risk | | low risk | low risk | unclear risk |
| Amin 2008 | | unclear risk | low risk | unclear risk | | low risk | low risk | low risk |
| Lo 2008 | | low risk | low risk | unclear risk | | low risk | low risk | low risk |
| Kumar 2009 | | low risk | low risk | low risk | | low risk | low risk | low risk |
| Lo 2009 | | low risk | low risk | low risk | | low risk | low risk | low risk |
| Garcı´a-Paga´n 2009 | | low risk | low risk | unclear risk | | low risk | low risk | unclear risk |
| Sarin 2010 | | unclear risk | low risk | low risk | | low risk | low risk | low risk |
| Mishra 2010 | | low risk | low risk | low risk | | low risk | low risk | low risk |
| Harras 2010 | | low risk | low risk | unclear risk | | low risk | unclear risk | low risk |
| Ljubicić 2011 | | unclear risk | low risk | low risk | | low risk | low risk | low risk |
| Kong 2015 | | low risk | low risk | unclear risk | | low risk | low risk | low risk |
| Ali 2016 | | low risk | low risk | unclear risk | | low risk | low risk | low risk |
| Lv 2017 | | low risk | low risk | low risk | | low risk | low risk | low risk |
| Mansour 2017 | | low risk | low risk | low risk | | low risk | low risk | low risk |
| Elsebaey 2019 | | low risk | low risk | low risk | | low risk | low risk | unclear risk |

**Supplementary Table 2.** The table of potential scale reduction factors for Brooks-Gelman-Rubin diagnostic plot. EVL, endoscopic variceal ligation; TIPS, transjugular intrahepatic portosystemic shunt; DSRS, distal splenorenal shunt; EIS, endoscopic injection sclerotherapy; ETA, endoscopic tissue.

| Comparsion | Point est. | Upper C.I. |
| --- | --- | --- |
| d.EVL.TIPS | 1 | 1 |
| d.EVL.medication | 1 | 1 |
| d.EVL.EVL + EIS | 1 | 1 |
| d.EVL.EIS | 1 | 1 |
| d.EVL.EVL + medication | 1 | 1 |
| d.EVL.ETA | 1 | 1 |
| d.EIS.DSRS | 1 | 1 |
| d.EIS.EIS + medication | 1 | 1 |
| sd.d | 1 | 1 |
| Multivariate psrf | 1 | 1 |
